# Metabolic remodeling and the modulatory role of vitamin D deficiency in African American children and adolescents with obesity

**DOI:** 10.1038/s41366-025-02003-0

**Published:** 2026-01-12

**Authors:** Hui-Qi Qu, John J. Connolly, Frank Mentch, Joseph Glessner, Hakon Hakonarson

**Affiliations:** 1 The Center for Applied Genomics, Children’s Hospital of Philadelphia, Philadelphia, PA USA; 2 Department of Pediatrics, The Perelman School of Medicine, University of Pennsylvania, Philadelphia, PA USA; 3 Division of Human Genetics, Children’s Hospital of Philadelphia, Philadelphia, PA USA; 4 Division of Pulmonary Medicine, Children’s Hospital of Philadelphia, Philadelphia, PA USA; 5 Faculty of Medicine, University of Iceland, Reykjavik, Iceland

**Keywords:** Translational research, Obesity

## Abstract

**Background:**

Obesity is a complex metabolic condition with a disproportionate impact among African American youth. Metabolomic profiling enables high-resolution mapping of obesity-related biochemical changes across metabolic pathways.

**Methods:**

We analyzed 551 African American pediatric participants (aged 2–21 years) recruited at the Children’s Hospital of Philadelphia between 2002 and 2020. Vitamin D deficiency was observed in 86 participants, including 62 of 309 individuals with overweight or obesity (20%). Plasma metabolites were quantified using the Nightingale NMR platform. Obesity was defined using age- and sex-specific BMI percentiles.

**Results:**

A total of 142 individual metabolite markers and ratios were significantly associated with obesity (FDR < 0.05). Obesity‑related metabolic changes were characterized by elevated branched-chain and aromatic amino acids, GlycA, and triglyceride-rich VLDL subclasses, along with reduced Gly, large HDL particles, and cholesterol-enriched lipoproteins. Global enrichment analysis identified a significant overrepresentation of nominally significant interactions with vitamin D deficiency (*p* < 2.2 × 10^−^¹⁶).

**Conclusions:**

Obesity in African American youth is linked to widespread metabolic remodeling across amino acid, lipid, and inflammatory pathways, reflecting core features of cardiometabolic risk. Vitamin D status may also influence these responses, suggesting a potential role in modifying obesity-related risk. These findings highlight the importance of early identification and prevention strategies and point to vitamin D as a possible target for future investigation.

## Introduction

Obesity is a multifactorial metabolic disorder associated with insulin resistance, dyslipidemia, systemic inflammation, and increased cardiometabolic risk [1]. Its pathophysiology extends beyond excess adiposity to involve complex interactions between genetic background, environmental exposures, and metabolic regulation [2]. Traditional biomarkers such as cholesterol levels and body mass index (BMI) offer clinical utility, but fail to capture the molecular complexity underlying obesity-related disease risk. This is particularly relevant in African American populations, who remain underrepresented in molecular studies despite a disproportionately high obesity burden [3].

Recent advances in high-throughput metabolomics have enabled a more granular dissection of metabolic phenotypes, offering insights into both disease mechanisms and potential therapeutic targets. The Nightingale nuclear magnetic resonance (NMR) metabolomics platform has emerged as a robust and reproducible tool for quantifying a wide array of circulating metabolites. This platform provides quantitative measurements of 249 metabolic biomarkers across 18 metabolite groups, including comprehensive coverage of lipoprotein subclasses, fatty acids, amino acids, and inflammation-related molecules [4, 5]. Its reproducibility and standardization make it particularly suited for large-scale population-based studies.

In pediatric cohorts, African American children and adolescents consistently exhibit higher rates of overweight and obesity compared to other racial and ethnic groups in the United States [6]. Furthermore, ancestry-related differences in lipid metabolism and inflammatory signaling have been observed [7], underscoring the need for ancestry-specific metabolic profiling. Vitamin D deficiency is frequently observed in children and adolescents with obesity [8, 9]. It is also common in African American populations, partly due to reduced skin synthesis and possibly diet and lifestyle [10]. Beyond its well-established role in calcium and bone metabolism, vitamin D has been implicated in pathways related to insulin sensitivity, lipid handling, and inflammation [11].

Although emerging evidence suggests that deficiency may exacerbate obesity-related disturbances [12], few studies have examined vitamin D as a potential modifier of obesity-related metabolism in African American youth. In this study, we applied the Nightingale NMR platform to a cohort of African American individuals recruited at the Center for Applied Genomics (CAG) at the Children’s Hospital of Philadelphia (CHOP). Our primary aim was to characterize the metabolomic architecture of obesity in this underrepresented group. Our exploratory aim was to assess whether vitamin D deficiency modifies obesity-related metabolic effects. Of the 249 biomarkers measured, 98 represented lipoprotein subclasses, 70 captured relative lipoprotein lipid concentrations, 18 reflected fatty acid profiles, and 9 encompassed phospholipids and other lipid species. In addition, metabolite ratios were computed in a data-driven manner between pairs of biomarkers with opposing directions of association (positive vs. negative coefficients for obesity) to capture integrated metabolic shifts that might be obscured at the individual marker level. We hypothesized that obesity would be associated with broad, coordinated alterations across metabolite classes, and that vitamin D deficiency might influence the magnitude or direction of these associations.

## Methods

### Subjects

All pediatric participants were recruited at CHOP-CAG between 2002 and 2020. African American ancestry was inferred using genome-wide SNP genotyping, with principal component analysis (PCA) applied as a complementary tool to assess population ancestry. Individuals whose PCA results indicated non-African American ancestry and were inconsistent with self-reported ancestry were excluded to ensure a genetically homogeneous study cohort. As CHOP sees patients up to the age of 21, pediatric status was defined as ≤21 years of age, also in accordance with the American Academy of Pediatrics [13]. Participants with known Mendelian disorders were excluded.

Body mass index (BMI) was used to classify overweight and obesity based on age-specific thresholds. This approach preserves clinically meaningful classification thresholds, provides an interpretable trend across categories, and reduces undue influence from extreme BMI values that can occur when modeling BMI as a continuous variable. For individuals aged 2 to 19 years, BMI percentile cutoffs from the Centers for Disease Control and Prevention (CDC) were applied [14, 15]:

- Overweight: ≥85th percentile and <95th percentile for age and sex;
- Class I obesity: ≥95th percentile and <120% of the 95th percentile;
- Class II obesity: ≥120% of the 95th percentile or BMI ≥ 35 kg/m², whichever was lower;
- Class III obesity: ≥140% of the 95th percentile or BMI ≥ 40 kg/m², whichever was lower.
- For participants aged >19 years, standard adult BMI cutoffs were used.

The final study cohort included plasma metabolomic analyses of 551 African American individuals (238 males, 313 females). Based on BMI classification, 125 were overweight, 88 had Class I obesity, 52 had Class II obesity, and 44 had Class III (extreme) obesity. Vitamin D deficiency was present in 86 participants. Among them, 62 individuals had both overweight or obesity and vitamin D deficiency: 15 in the overweight group, 17 in Class I, 11 in Class II, and 19 in Class III. A total of 218 individuals had neither obesity nor vitamin D deficiency.

### Metabolomic profiling

Plasma samples (350 μL per subject) were collected in ethylenediaminetetraacetic acid (EDTA)-anticoagulated tubes, processed within 2 h, and stored at −80 °C until analysis. Metabolomic profiling was performed using Nightingale Health’s high-throughput proton ^1^H NMR spectroscopy platform (version 2020) [16]. The acquisition protocol included a water-suppressed one-dimensional (1D) ^1^H spectrum, acquired using nuclear Overhauser effect spectroscopy with presaturation (NOESY-presat), for quantification of low-molecular-weight metabolites and a T_2_-filtered Carr–Purcell–Meiboom–Gill (CPMG) experiment for lipid and lipoprotein subclass profiling. Spectral regions between 0.2–9.0 ppm were analyzed, with residual water (4.5–5.0 ppm) and EDTA signals excluded. Biomarker concentrations were derived from validated regression models calibrated against chemical reference methods under ISO-certified quality management [4, 5]. Sample quality was routinely assessed using Nightingale’s automated QC system to ensure data reliability.

### Data analysis

Raw metabolomic data were imported into R (version 4.4.1) [17]. All metabolite concentrations were log₂-transformed using a small constant (1e−9) to handle zero values, followed by *z*-score normalization (mean-centering and unit variance scaling) to ensure comparability across features.

Correlation analyses were conducted in Python using the Pandas, NumPy, and Statsmodels libraries [18]. Obesity status was encoded as an ordinal variable (0–4) representing increasing severity (without obesity, overweight, Class I–III obesity), and vitamin D deficiency was treated as a binary variable (deficiency vs. non-deficiency). An interaction term (obesity × vitamin D deficiency) was created to evaluate joint effects. Initial univariate analyses assessed associations between individual metabolites and obesity or vitamin D deficiency. Ordinary least squares (OLS) regression was used for obesity as a continuous ordinal outcome; logistic regression was used for vitamin D deficiency. Each model included a single metabolite as the primary predictor, with age, sex, and the alternate phenotype (e.g., obesity status when modeling vitamin D) included as covariates. Metabolites were excluded if the outcome showed insufficient variability within the subgroup to ensure model stability.

To explore biologically meaningful relationships, additional metabolite ratios were computed between pairs with opposing directions of association. These derived features were re-analyzed using the same regression models to identify composite markers with stronger or more consistent associations. Interaction effects between obesity and vitamin D deficiency were evaluated using linear regression. Each model included obesity, vitamin D deficiency, their interaction term, and covariates (age and sex). All statistical significance was adjusted using the Benjamini–Hochberg false discovery rate (FDR), correcting for multiple testing across individual metabolites and derived ratios.

A logistic regression model was fitted to examine the association between obesity and vitamin D deficiency, adjusting for age and sex. The analysis used the glm() function in R with a binomial family. Statistical significance was determined using a two-sided *p* value threshold of 0.05.

**Fig. 1:**
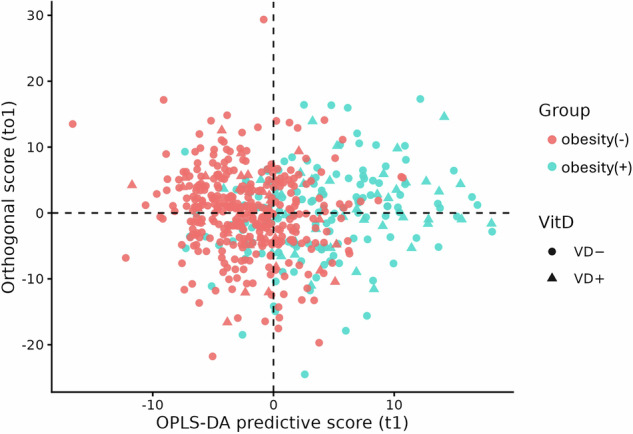
OPLS-DA scores plot (without obesity vs. with obesity). Scores from orthogonal projections to latent structures discriminant analysis (OPLS-DA) with one predictive component are shown (*x*-axis: predictive score t1; *y*-axis: first orthogonal score to1). Each point represents one participant, colored by obesity group (without obesity = normal-weight + overweight; with obesity = Class I–III). Shapes indicate vitamin D deficiency status (VD− = no deficiency, VD+ = deficiency). Greater separation along the *x*-axis reflects stronger discrimination between participants without obesity and those with obesity.

To visualize multivariate and correlational structure, we additionally performed orthogonal projections to latent structures–discriminant analysis (OPLS-DA) using the ropls package [19]. OPLS-DA scores plot was used to examine separation between participants with obesity and participants without obesity, and to assess potential modulation by vitamin D deficiency (Fig. 1). Heatmaps generated with the pheatmap package [20] were created to illustrate metabolite-level clustering and intercorrelation patterns by molecular class (Supplementary Files S1 and S2). PCA was additionally applied to the normalized NMR metabolome to explore potential age-related variation (Supplementary File S3).

### Ethical approval

This study was approved by the Institutional Review Board (IRB) of the Children’s Hospital of Philadelphia. Human participants and personal information were encrypted to ensure that no PHI was included and that study participants were de-identified. All human subjects or their proxies provided written informed consent. All methods were performed in accordance with the relevant guidelines and regulations.

## Results

### BMI classification and metabolomic markers

A total of 140 metabolite markers and 2 marker ratios were significantly correlated with BMI classifications (FDR < 0.05) (Table 1 and Supplementary Table 1). Multivariate visualization by OPLS-DA confirmed a clear separation between participants with obesity and those without obesity, along the first predictive component (Fig. 1). To complement single-marker analyses, phenotype-ordered heatmaps were constructed for each metabolite class (Supplementary File S1). These visualizations revealed coherent, class-specific clustering patterns. Pairwise Spearman correlation heatmaps (Supplementary File S2) further highlighted modular organization of co-regulated metabolites. These results reflect widespread metabolic changes across lipid transport, lipoprotein composition, amino acid metabolism, and inflammatory activity in relation to BMI classification. PCA of the normalized NMR metabolome showed no distinct clustering by age or pediatric age group, indicating that age contributed modestly to global metabolic variance after accounting for obesity (Supplementary File S3).

**Table 1:** 140 Metabolite markers and 2 ratios correlated with BMI classifications (FDR < 0.05).

Marker	Group	Coefficient	*p* value	FDR
Gly	Amino acids	−0.3111	3.73E−09	9.77E−07
Tyr	Amino acids	0.279	1.62E−07	3.12E−05
Val	Amino acids	0.2377	5.21E−06	6.95E−04
Total_BCAA	Amino acids	0.2131	4.98E−05	5.78E−03
His	Amino acids	−0.2149	9.00E−05	9.80E−03
ApoB	Apolipoproteins	0.2972	3.03E−08	6.65E−06
ApoA1	Apolipoproteins	−0.261	2.29E−07	4.29E−05
HDL_C	Cholesterol	−0.3424	1.17E−11	4.76E−09
VLDL_C	Cholesterol	0.3521	7.49E−11	2.55E−08
Remnant_C	Cholesterol	0.2638	9.07E−07	1.41E−04
non_HDL_C	Cholesterol	0.2382	6.56E−06	8.56E−04
LDL_C	Cholesterol	0.2088	6.15E−05	6.96E−03
Clinical_LDL_C	Cholesterol	0.2086	6.82E−05	7.60E−03
HDL_CE	Cholesteryl esters	−0.3566	1.74E−12	8.58E−10
VLDL_CE	Cholesteryl esters	0.3345	6.53E−10	1.94E−07
LDL_CE	Cholesteryl esters	0.2188	2.57E−05	3.07E−03
MUFA_pct	Fatty acids	0.3163	5.00E−09	1.23E−06
PUFA_by_MUFA	Fatty acids	−0.285	5.60E−08	1.14E−05
Omega_6_pct	Fatty acids	−0.2298	6.60E−06	8.56E−04
MUFA	Fatty acids	0.2359	7.20E−06	9.25E−04
PUFA_pct	Fatty acids	−0.2028	5.38E−05	6.14E−03
VLDL_FC	Free cholesterol	0.375	3.87E−12	1.72E−09
HDL_FC	Free cholesterol	−0.2851	1.66E−08	3.81E−06
GlycA	Inflammation	0.3724	1.11E−12	5.64E−10
VLDL_P	Lipoprotein particle concentrations	0.3623	2.69E−11	1.01E−08
LDL_P	Lipoprotein particle concentrations	0.2946	3.68E−08	7.72E−06
HDL_P	Lipoprotein particle concentrations	−0.1772	4.10E−04	0.0412
HDL_size	Lipoprotein particle sizes	−0.5136	1.34E−22	6.37E−19
VLDL_size	Lipoprotein particle sizes	0.4156	2.00E−15	1.90E−12
L_HDL_CE	Lipoprotein subclasses	−0.5624	3.36E−24	4.80E−20
L_HDL_C	Lipoprotein subclasses	−0.5517	4.95E−23	3.53E−19
L_HDL_P	Lipoprotein subclasses	−0.5034	4.18E−21	1.49E−17
L_HDL_L	Lipoprotein subclasses	−0.4907	1.91E−20	5.46E−17
L_HDL_FC	Lipoprotein subclasses	−0.5336	2.56E−18	4.79E−15
L_HDL_PL	Lipoprotein subclasses	−0.4977	2.69E−18	4.79E−15
L_VLDL_L	Lipoprotein subclasses	0.4624	2.38E−18	4.79E−15
XL_VLDL_L	Lipoprotein subclasses	0.4474	2.03E−17	2.90E−14
L_VLDL_TG	Lipoprotein subclasses	0.5011	1.21E−16	1.57E−13
L_VLDL_FC	Lipoprotein subclasses	0.4285	3.82E−16	4.55E−13
L_VLDL_P	Lipoprotein subclasses	0.4281	7.64E−16	8.39E−13
XL_HDL_CE	Lipoprotein subclasses	−0.461	8.13E−15	7.25E−12
XL_VLDL_P	Lipoprotein subclasses	0.4094	1.43E−14	1.20E−11
L_VLDL_C	Lipoprotein subclasses	0.4024	2.70E−14	2.03E−11
XL_VLDL_C	Lipoprotein subclasses	0.4027	2.91E−14	2.07E−11
XL_VLDL_CE	Lipoprotein subclasses	0.3852	2.32E−13	1.56E−10
M_VLDL_TG	Lipoprotein subclasses	0.3748	6.22E−13	3.55E−10
XL_HDL_L	Lipoprotein subclasses	−0.3973	8.66E−13	4.57E−10
L_VLDL_CE	Lipoprotein subclasses	0.373	2.15E−12	1.02E−09
XL_HDL_C	Lipoprotein subclasses	−0.4261	3.76E−12	1.72E−09
S_VLDL_P	Lipoprotein subclasses	0.3783	6.08E−12	2.63E−09
S_VLDL_L	Lipoprotein subclasses	0.3749	7.24E−12	3.04E−09
S_VLDL_CE	Lipoprotein subclasses	0.3691	1.40E−11	5.54E−09
M_VLDL_L	Lipoprotein subclasses	0.3488	4.50E−11	1.61E−08
XL_VLDL_FC	Lipoprotein subclasses	0.3437	6.75E−11	2.35E−08
XL_HDL_P	Lipoprotein subclasses	−0.3867	8.30E−11	2.75E−08
S_VLDL_C	Lipoprotein subclasses	0.3518	9.87E−11	3.20E−08
S_HDL_TG	Lipoprotein subclasses	0.3428	1.75E−10	5.53E−08
S_VLDL_PL	Lipoprotein subclasses	0.3403	3.34E−10	1.04E−07
M_VLDL_P	Lipoprotein subclasses	0.3346	4.79E−10	1.46E−07
M_HDL_CE	Lipoprotein subclasses	−0.3002	3.34E−09	9.00E−07
XL_VLDL_PL	Lipoprotein subclasses	0.3099	4.67E−09	1.17E−06
M_VLDL_PL	Lipoprotein subclasses	0.3054	7.80E−09	1.89E−06
S_VLDL_FC	Lipoprotein subclasses	0.3101	9.59E−09	2.28E−06
M_HDL_C	Lipoprotein subclasses	−0.2883	1.23E−08	2.87E−06
S_LDL_TG	Lipoprotein subclasses	0.3004	2.76E−08	6.26E−06
XL_VLDL_TG	Lipoprotein subclasses	0.2664	2.91E−08	6.48E−06
M_VLDL_FC	Lipoprotein subclasses	0.2973	3.15E−08	6.82E−06
L_LDL_P	Lipoprotein subclasses	0.2956	3.32E−08	7.06E−06
M_LDL_P	Lipoprotein subclasses	0.2934	3.95E−08	8.17E−06
S_VLDL_TG	Lipoprotein subclasses	0.2961	6.23E−08	1.25E−05
M_HDL_P	Lipoprotein subclasses	−0.2697	9.12E−08	1.78E−05
XS_VLDL_FC	Lipoprotein subclasses	0.2738	2.51E−07	4.58E−05
XS_VLDL_P	Lipoprotein subclasses	0.2783	2.81E−07	5.08E−05
L_VLDL_PL	Lipoprotein subclasses	0.2553	3.22E−07	5.75E−05
XS_VLDL_L	Lipoprotein subclasses	0.2765	3.36E−07	5.92E−05
M_HDL_L	Lipoprotein subclasses	−0.2574	3.45E−07	5.93E−05
XXL_VLDL_CE	Lipoprotein subclasses	0.2751	3.43E−07	5.93E−05
M_LDL_TG	Lipoprotein subclasses	0.2711	3.53E−07	5.99E−05
XL_HDL_PL	Lipoprotein subclasses	−0.3125	4.70E−07	7.70E−05
XXL_VLDL_P	Lipoprotein subclasses	0.2642	5.37E−07	8.61E−05
M_LDL_CE	Lipoprotein subclasses	0.2584	5.89E−07	9.24E−05
M_HDL_PL	Lipoprotein subclasses	−0.2481	9.98E−07	1.53E−04
L_LDL_TG	Lipoprotein subclasses	0.2577	1.25E−06	1.89E−04
S_LDL_P	Lipoprotein subclasses	0.2597	1.29E−06	1.93E−04
S_LDL_L	Lipoprotein subclasses	0.255	1.33E−06	1.98E−04
M_LDL_L	Lipoprotein subclasses	0.2503	1.41E−06	2.07E−04
S_LDL_CE	Lipoprotein subclasses	0.2517	1.74E−06	2.53E−04
S_LDL_PL	Lipoprotein subclasses	0.2526	1.91E−06	2.72E−04
M_LDL_PL	Lipoprotein subclasses	0.2469	1.94E−06	2.74E−04
M_LDL_C	Lipoprotein subclasses	0.2455	2.21E−06	3.07E−04
XXL_VLDL_C	Lipoprotein subclasses	0.2541	2.21E−06	3.07E−04
XS_VLDL_C	Lipoprotein subclasses	0.2493	3.79E−06	5.15E−04
S_LDL_C	Lipoprotein subclasses	0.2409	4.54E−06	6.10E−04
M_VLDL_C	Lipoprotein subclasses	0.2449	6.35E−06	8.39E−04
IDL_TG	Lipoprotein subclasses	0.2422	7.53E−06	9.60E−04
M_HDL_FC	Lipoprotein subclasses	−0.218	9.76E−06	1.22E−03
XS_VLDL_TG	Lipoprotein subclasses	0.2373	1.51E−05	1.85E−03
IDL_P	Lipoprotein subclasses	0.2318	1.54E−05	1.88E−03
XS_VLDL_CE	Lipoprotein subclasses	0.2293	2.15E−05	2.59E−03
XS_VLDL_PL	Lipoprotein subclasses	0.1983	4.36E−05	5.09E−03
XXL_VLDL_L	Lipoprotein subclasses	0.2129	5.03E−05	5.79E−03
XXL_VLDL_PL	Lipoprotein subclasses	0.2121	6.23E−05	6.99E−03
M_LDL_FC	Lipoprotein subclasses	0.2057	7.96E−05	8.81E−03
L_LDL_PL	Lipoprotein subclasses	0.2057	8.57E−05	9.41E−03
S_LDL_FC	Lipoprotein subclasses	0.2018	1.21E−04	0.0129
L_LDL_L	Lipoprotein subclasses	0.1992	1.35E−04	0.0143
L_LDL_CE	Lipoprotein subclasses	0.1971	1.51E−04	0.0159
L_LDL_C	Lipoprotein subclasses	0.1884	3.04E−04	0.0312
M_VLDL_CE	Lipoprotein subclasses	0.2262	3.23E−04	0.0329
IDL_PL	Lipoprotein subclasses	0.192	3.32E−04	0.0336
TG_by_PG	Other lipids	0.4456	7.98E−18	1.26E−14
VLDL_PL	Phospholipids	0.3919	4.75E−13	2.95E−10
HDL_PL	Phospholipids	−0.2977	4.35E−09	1.11E−06
LDL_PL	Phospholipids	0.2271	1.45E−05	1.80E−03
L_HDL_CE_pct	Relative lipoprotein lipid concentrations	−0.6231	2.40E−13	1.56E−10
L_HDL_C_pct	Relative lipoprotein lipid concentrations	−0.6828	8.07E−13	4.43E−10
XL_HDL_TG_pct	Relative lipoprotein lipid concentrations	0.3728	1.93E−11	7.46E−09
S_HDL_TG_pct	Relative lipoprotein lipid concentrations	0.3525	3.36E−11	1.23E−08
XL_HDL_C_pct	Relative lipoprotein lipid concentrations	0.3478	9.63E−10	2.80E−07
M_HDL_C_pct	Relative lipoprotein lipid concentrations	−0.3295	1.01E−09	2.88E−07
L_LDL_FC_pct	Relative lipoprotein lipid concentrations	−0.3226	1.22E−09	3.35E−07
M_HDL_CE_pct	Relative lipoprotein lipid concentrations	−0.3208	3.76E−09	9.77E−07
M_HDL_PL_pct	Relative lipoprotein lipid concentrations	0.2745	6.86E−08	1.36E−05
M_LDL_FC_pct	Relative lipoprotein lipid concentrations	−0.2777	2.09E−07	3.98E−05
XL_HDL_FC_pct	Relative lipoprotein lipid concentrations	0.2369	2.42E−07	4.48E−05
M_LDL_CE_pct	Relative lipoprotein lipid concentrations	0.2563	4.20E−07	7.06E−05
XL_VLDL_CE_pct	Relative lipoprotein lipid concentrations	−0.2653	4.39E−07	7.29E−05
M_VLDL_CE_pct	Relative lipoprotein lipid concentrations	−0.4099	5.47E−07	8.66E−05
S_HDL_PL_pct	Relative lipoprotein lipid concentrations	−0.261	1.78E−06	2.56E−04
XL_VLDL_C_pct	Relative lipoprotein lipid concentrations	−0.2442	3.21E−06	4.41E−04
M_HDL_TG_pct	Relative lipoprotein lipid concentrations	0.2289	9.18E−06	1.16E−03
L_HDL_PL_pct	Relative lipoprotein lipid concentrations	0.6495	3.94E−05	4.65E−03
M_VLDL_C_pct	Relative lipoprotein lipid concentrations	−0.2183	9.30E−05	0.01
L_HDL_TG_pct	Relative lipoprotein lipid concentrations	0.1946	2.72E−04	0.0285
M_LDL_TG_pct_by_L_HDL_PL	Relative lipoprotein lipid concentrations/Lipoprotein subclasses	−0.0114	2.76E−04	0.0287
XL_VLDL_TG_pct_by_L_HDL_TG	Relative lipoprotein lipid concentrations/Lipoprotein subclasses	0.0278	2.82E−04	0.0291
VLDL_L	Total lipids	0.4117	2.25E−14	1.78E−11
HDL_L	Total lipids	−0.3075	1.19E−09	3.32E−07
LDL_L	Total lipids	0.2195	2.58E−05	3.07E−03
VLDL_TG	Triglycerides	0.4308	1.35E−15	1.38E−12
Total_TG	Triglycerides	0.3882	6.02E−13	3.55E−10
LDL_TG	Triglycerides	0.2679	5.00E−07	8.11E−05

#### Pro-atherogenic lipid profile

For apolipoproteins, ApoB showed a positive association with BMI classification, while ApoA1 was negatively associated. In the cholesterol group, positive associations were observed for VLDL_C, Remnant_C, non_HDL_C, LDL_C, and Clinical_LDL_C, whereas HDL_C was negatively associated. Cholesteryl esters showed similar patterns: VLDL_CE and LDL_CE were positively associated with BMI, while HDL_CE was negatively associated.

Fatty acid profiles differed by BMI status, with positive associations for MUFA and MUFA_pct, and negative associations for PUFA_pct, PUFA_by_MUFA, and Omega_6_pct. In the free cholesterol group, VLDL_FC was positively associated and HDL_FC was negatively associated with BMI.

Markers from other lipid-related categories were also significant. In phospholipids, VLDL_PL and LDL_PL were positively associated, while HDL_PL showed a negative association. In the triglyceride group, positive associations were observed for VLDL_TG, Total_TG, and LDL_TG. The ratio TG_by_PG was also positively associated with BMI.

#### Lipoprotein remodeling and particle size

Among lipoprotein particle concentrations, VLDL_P and LDL_P were positively associated, while HDL_P was negatively associated. For particle sizes, VLDL_size was positively associated, and HDL_size was negatively associated with BMI.

Strong and consistent patterns were observed in lipoprotein subclasses. VLDL-related markers including L_VLDL_L, XL_VLDL_L, L_VLDL_TG, L_VLDL_FC, L_VLDL_P, and XL_VLDL_P were all positively associated with BMI. In contrast, HDL-related subclasses such as L_HDL_CE, L_HDL_C, L_HDL_P, L_HDL_L, L_HDL_FC, and L_HDL_PL showed strong negative associations.

For relative lipoprotein lipid concentrations, L_HDL_C_pct and L_HDL_CE_pct were negatively associated with BMI, while L_HDL_PL_pct, XL_HDL_TG_pct, S_HDL_TG_pct, and XL_HDL_C_pct were positively associated. Additional trends included negative associations for M_HDL_C_pct, L_LDL_FC_pct, and M_HDL_CE_pct, and positive associations for M_HDL_PL_pct and M_LDL_CE_pct. M_VLDL_CE_pct and XL_VLDL_CE_pct were negatively associated with BMI, while XL_HDL_FC_pct and M_HDL_TG_pct were positively associated.

Notably, the largest effect sizes (|*β*| ≥ 0.40) were concentrated in HDL- and VLDL-related traits. Strong negative *β* values for large HDL subclasses and compositional measures (e.g., L_HDL_C_pct, L_HDL_CE_pct, HDL_size) highlight substantial depletion of cholesterol- and phospholipid-rich HDL particles, whereas strong positive *β* values for VLDL measures (e.g., L_VLDL_TG, VLDL_TG, VLDL_size) indicate pronounced enrichment of triglyceride-rich VLDL particles. Additional large effects in compositional markers such as L_HDL_PL_pct, TG_by_PG, and M_VLDL_CE_pct point to qualitative remodeling of lipoprotein lipid content. Together, these large-effect markers emphasize a systemic shift from cardioprotective HDL toward atherogenic VLDL dominance.

#### Reduced protective amino acids and systemic inflammation

Among amino acids, BMI classification was positively associated with Tyr, Val, and Total_BCAA, and negatively associated with Gly and His, indicating shifts in amino acid metabolism related to BMI. The inflammation marker GlycA also showed a strong positive correlation with BMI.

### Vitamin D deficiency’s interaction with obesity

#### Exploratory evidence of global enrichment

**Fig. 2:**
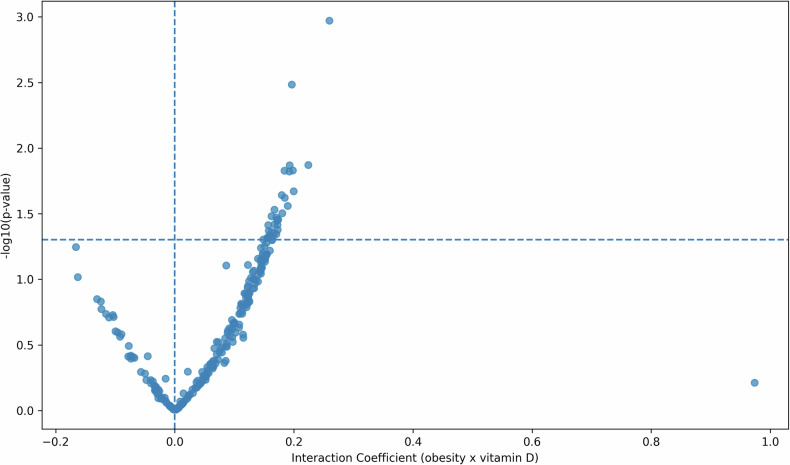
Volcano plot of obesity x vitamin D deficiency interaction effects on plasma metabolites. The horizontal dashed line indicates nominal significance (*p* = 0.05). A subset of metabolites appeared in the upper right quadrant, indicating positive interaction effects (stronger associations with obesity under vitamin D deficiency). No metabolites reached significance in the negative interaction quadrant, suggesting a directional bias toward amplification rather than attenuation of obesity-related metabolic changes. The *x*-axis shows the regression coefficient of the interaction term, and the *y*-axis shows −log_10_(*p* value).

When assessing the correlation between metabolite markers and vitamin D deficiency, adjusting for age, sex, and obesity, no single marker passed the FDR threshold for significance (summarized in Table 2; full results in Supplementary Table 2). Similarly, no individual interaction effects between obesity and vitamin D deficiency on metabolite levels remained significant after FDR correction (summarized in Table 2; full results in Supplementary Table 3). However, at a global level, the number of nominally significant interaction terms was higher than expected by chance. Out of 11,057 metabolite markers/ratios tested, 879 showed nominal significance, compared to ~553 expected under the null hypothesis (*α* = 0.05). A binomial test confirmed that this enrichment was highly significant (*p* < 2.2 × 10^−^¹⁶). Interaction analyses were summarized in a volcano plot contrasting regression coefficients and significance values for obesity x vitamin D deficiency terms (Fig. 2). These findings should be viewed as exploratory, suggesting a potential distributed influence of vitamin D deficiency on obesity-related metabolic pathways that warrants further investigation.

**Table 2:** Summary of metabolomic associations with obesity and vitamin D deficiency.

Analysis	Key findings	Notes
Obesity main effects (Supplementary Table 1)	Significant associations across lipoproteins, fatty acids, amino acids, and inflammation	142 total markers (FDR < 0.05): 25 large effects ( |*β*| ≥ 0.40); 108 medium effects: (0.20 ≤ |*β* | <0.40); 9 small effects: |*β*| < 0.20.
Vitamin D deficiency main effects (Supplementary Table 2)	No metabolites significant after FDR correction	Indicates no strong independent effects after adjustment for age, sex, and obesity.
Obesity × Vitamin D deficiency interactions (Supplementary Table 3)	No single interaction significant after FDR correction, but strong enrichment signal	879 of 11,057 markers/ratios nominally significant (vs. ~553 expected under null), binomial *p* < 2.2 × 10^−^¹⁶. Six metabolite ratios showed nominal interaction effects and associations with vitamin D deficiency. Findings exploratory.

#### Specific metabolite ratios

Among the 879 nominally significant interactions, six metabolite ratios showed both interaction effects (*p* < 0.05, all with negative coefficients) and associations with vitamin D deficiency (p < 0.05, also with negative coefficients). These included VLDL_TG_by_M_HDL_C, TG_by_PG_by_M_HDL_C, S_HDL_TG_by_M_HDL_C, M_LDL_P_by_S_LDL_C_pct, S_LDL_CE_by_S_LDL_C_pct, and S_LDL_P_by_S_LDL_C_pct. The negative interaction coefficients indicate that the effect of obesity on these ratios may be diminished in individuals with vitamin D deficiency. While none of these ratios are significantly correlated with obesity after correction, they highlight potential areas for future study.

## Discussion

### Obesity and metabolomic markers

**Fig. 3:**
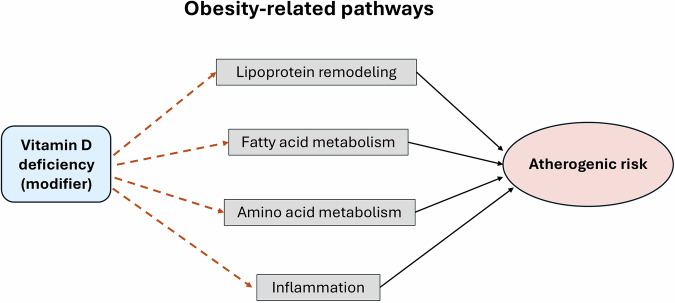
The potential modulatory role of vitamin D deficiency in obesity-related metabolomic remodeling. Obesity is the primary driver, inducing coordinated alterations across four key metabolic pathways: lipoprotein remodeling, fatty acid shifts, amino acid imbalance, and systemic inflammation. These pathways converge on an adverse outcome defined by an atherogenic and insulin-resistant metabolic profile. Vitamin D deficiency acts as a context-dependent modifier, influencing each pathway through distinct mechanisms: (i) impaired regulation of hepatic lipid metabolism and lipoprotein enzymes (LPL, CETP), aggravating VLDL enrichment and HDL dysfunction; (ii) reduced control of lipid synthesis and fatty acid oxidation, promoting metabolic inflexibility; (iii) diminished effects on insulin sensitivity and mitochondrial function, amplifying BCAA accumulation and Gly depletion; and (iv) loss of anti-inflammatory signaling, contributing to systemic inflammation. Together, these interactions suggest that vitamin D deficiency may amplify obesity-related metabolic risk.

Our results demonstrate a distinct and comprehensive metabolomic signature associated with BMI classifications, comprising 140 metabolite markers and 2 marker ratios spanning amino acids, lipid species, lipoprotein subclasses, and inflammatory markers (Box 1 and Fig. 3). The breadth and consistency of these associations underscore the systemic and integrated nature of obesity-related metabolic dysregulation.

#### Pro-atherogenic lipid profile

Lipidomic alterations add another layer of complexity. The combination of elevated ApoB and suppressed ApoA1 reflects a well-characterized atherogenic profile [21]. Increases in VLDL_C, LDL_C, and Remnant_C, along with reductions in HDL_C, recapitulate the classical dyslipidemia of obesity [22]. However, subclass-level analysis reveals a deeper remodeling: obesity is strongly associated with enrichment of VLDL subclasses (L_VLDL_P, XL_VLDL_P, L_VLDL_TG) and concurrent depletion of large HDL particles (L_HDL_C, L_HDL_CE, L_HDL_P). These coordinated shifts suggest a metabolic state characterized by increased hepatic VLDL production [23] and impaired HDL maturation, a profile known to promote atherosclerosis and impair reverse cholesterol transport [24].

Fatty acid perturbations, including elevated MUFA and reduced PUFA_pct, PUFA_by_MUFA, and Omega_6_pct, further support a lipogenic phenotype associated with obesity, consistent with hepatic steatosis, metabolic inflexibility, and proinflammatory lipid signaling [25].

#### Lipoprotein remodeling and particle size

Beyond individual markers, the coordinated behavior of lipoprotein subclasses reveals deeper systems-level insights. The uniformly negative correlations across large HDL subclasses, including cholesterol, cholesteryl esters, phospholipids, and free cholesterol, suggest a global depletion of functional HDL particles [26]. This pattern likely reflects defects in HDL biogenesis or accelerated catabolism, which together contribute to HDL dysfunction. In contrast, the consistent positive associations across VLDL subclasses, including triglycerides, CE, FC, PL, and particle number, point to a systemic upregulation of VLDL synthesis, highlighting hepatic overdrive as a central feature of the obesity-associated metabolome [23]. Importantly, the strongest effect sizes in our dataset ( | *β* | ≥ 0.40) were clustered in these pathways, with large negative *β* values for HDL traits (e.g., L_HDL_C_pct, L_HDL_CE_pct, HDL_size) and large positive *β* values for VLDL traits (e.g., L_VLDL_TG, VLDL_TG, VLDL_size). These high-magnitude markers emphasize not only the loss of HDL functionality [26, 27] but also the hepatic overproduction of triglyceride-rich VLDL particles [23], both of which are central drivers of obesity-related cardiometabolic risk. Additional compositional changes (e.g., L_HDL_PL_pct, TG_by_PG, M_VLDL_CE_pct) suggest qualitative remodeling of lipoproteins, further supporting the biological plausibility of our findings.

These subclass-wide patterns emphasize that obesity-related metabolic dysfunction cannot be reduced to isolated biomarkers. Instead, they reflect deeply integrated remodeling of lipoprotein architecture and systemic metabolic regulation. Recognizing and targeting these coordinated alterations may open the door to more effective biomarker discovery and therapeutic strategies aimed at restoring metabolic homeostasis.

#### Reduced protective amino acids and systemic inflammation

The observed amino acid profile, marked by elevated Total_BCAA, Val, and Tyr, and reduced Gly and His, mirrors known signatures of insulin resistance [28] and early-stage cardiometabolic dysfunction [29]. Of particular interest is the robust inverse association of Gly, which supports growing evidence that its depletion reflects mitochondrial overload and impaired oxidative metabolism, positioning Gly as a sensitive indicator of metabolic strain in obesity [30].

Elevated GlycA levels provide additional evidence of immunometabolic activation. As a composite NMR marker of systemic inflammation, GlycA integrates signals from multiple acute-phase proteins, reinforcing the inflammatory nature of obesity at the metabolic level [31].

Mechanistically, the amino-acid pattern observed here is consistent with disrupted substrate handling in pediatric obesity. Elevated BCAAs and Tyr likely result from reduced peripheral BCAA catabolism (e.g., decreased BCKDH activity via inhibitory phosphorylation) and mitochondrial stress, leading to accumulation of branched-chain ketoacids and acylcarnitines that impair insulin signaling and activate mTORC1 pathways, thereby driving hepatic lipogenesis and VLDL overproduction [26, 27]. Concurrent glycine depletion may arise from its diversion toward glutathione synthesis and acyl-glycine conjugation during lipid overload, as well as constrained one-carbon/folate flux, processes that parallel mitochondrial stress and the inflammatory milieu reflected by GlycA [28, 31]. Reduced histidine availability may further limit carnosine formation, diminishing buffering and anti-glycation capacity and thereby amplifying oxidative and inflammatory stress [32]. These amino-acid alterations link impaired oxidative metabolism to both insulin resistance and the lipoprotein remodeling observed in this cohort (VLDL enrichment and HDL depletion), suggesting coordinated disruption across amino-acid, redox, and lipid pathways in pediatric obesity.

Taken together, our findings provide a high-resolution map of obesity’s metabolomic footprint, uncover previously underappreciated structural lipid alterations, and lay a foundation for mechanistic and translational studies focused on obesity-related disease progression.

Box 1 Obesity and vitamin D influence on the circulating metabolomic markers **Obesity-driven metabolic alterations**

- ↑ Branched-chain and aromatic amino acids (Total_BCAA, Val, Tyr)
- ↓ Glycine and histidine → Mitochondrial stress, insulin resistance
- ↑ GlycA → Systemic low-grade inflammation
- ↑ ApoB, ↓ ApoA1 → Atherogenic shift in apolipoproteins
- ↑ VLDL_C, LDL_C, Remnant_C, ↓ HDL_C → Dyslipidemia

**Lipoprotein remodeling**

- ↑ VLDL subclasses (TG, CE, FC, PL, particle concentration) → Hepatic VLDL overproduction
- ↓ Large HDL subclasses (CE, FC, PL, particle size) → Impaired HDL maturation and function
- Altered lipid composition: ↑ TG-rich particles, ↓ CE- and PL-rich HDL → Lipoprotein dysfunction beyond concentration changes
- Largest effect sizes ( | *β* | ≥ 0.40) cluster in HDL depletion and VLDL enrichment → Strongest contributors to cardiometabolic risk

**Fatty acid shifts**

- ↑ MUFA, ↓ PUFA_pct, PUFA_by_MUFA, Omega_6_pct → Metabolic inflexibility, lipogenic signature

**Vitamin D deficiency**

- No individual metabolite associations after FDR correction
- Significant global enrichment of obesity × vitamin D interactions (879/11,057 markers, *p* < 2.2e–16) → Vitamin D acts as a modulator of obesity-related metabolic pathways

### Vitamin D deficiency as a modifier of obesity-associated metabolic profiles

#### Broad influence of vitamin D deficiency

In our study cohort, 72% of individuals with vitamin D deficiency were classified as overweight or with obesity, and logistic regression demonstrated that obesity was an independent predictor of vitamin D deficiency (*β* = 0.439, *p* < 0.001). After adjusting for age and sex, individuals with obesity had approximately 55% higher odds of vitamin D deficiency compared to those without obesity. This finding is consistent with prior evidence implicating adiposity in altered vitamin D storage, sequestration, or metabolism [12]. Despite this clinical overlap, no individual metabolite markers showed significant associations with vitamin D deficiency after FDR correction, controlling for age, sex, and obesity. Likewise, no single interaction term between obesity and vitamin D deficiency passed the threshold for multiple testing correction. However, the lack of individually significant markers does not imply the absence of biologically relevant effects. A global enrichment analysis revealed a statistically significant excess of nominally significant interaction terms (*p* < 0.05), with 879 out of 11,057 markers exceeding the expected rate under the null hypothesis (*p* < 2.2 × 10^−^¹⁶). This enrichment should be viewed as exploratory and hypothesis-generating. Vitamin D deficiency may exert a broad but subtle influence on the metabolome in the context of obesity, manifesting as distributed, small-effect interactions that are not detectable through conventional univariate approaches alone.

#### Vitamin D as a metabolic modifier

These results challenge the prevailing view of vitamin D deficiency as merely a downstream consequence of obesity. Instead, they support a more nuanced model in which vitamin D status modulates the metabolic consequences of obesity. In this framework, vitamin D deficiency may alter metabolic responses linked to excess adiposity, potentially affecting lipid metabolism, inflammation, or insulin sensitivity, supported by studies [33, 34]. For instance, the ratio of VLDL particle size to the cholesterol-to-total lipid ratio in small LDL (VLDL_size / S_LDL_C_pct) reflects the balance between hepatic expansion of triglyceride-rich VLDL particles [35] and the formation of dense, cholesterol-rich small LDL, a lipid pattern commonly seen in insulin resistance and atherogenic dyslipidemia [36]. While obesity alone does not significantly affect this ratio, vitamin D deficiency modifies the response: the significant negative interaction (*β* = –2.08, *p* = 4.01 × 10^−^⁵) indicates that obesity lowers this ratio only in the context of vitamin D deficiency. This suggests that vitamin D status alters how lipid pathways respond to adiposity, possibly by reducing hepatic triglyceride packaging into VLDL or enhancing the conversion of VLDL to dense LDL via altered lipolysis or lipid transfer.

Our findings indicate that the metabolic signature of obesity is dominant and may mask direct associations with vitamin D deficiency at the single-metabolite level. Rather than exerting large, independent effects, vitamin D deficiency likely modulates obesity-related metabolic pathways through subtle, system-level mechanisms. Such distributed influences, potentially involving lipid remodeling, insulin sensitivity, and inflammatory tone, may alter how metabolic networks respond to adiposity rather than shifting individual metabolite concentrations. These small, coordinated effects become apparent only when examining pathway-level enrichment or interaction patterns, as observed in our global analyses. This interpretation aligns with emerging evidence that vitamin D signaling interfaces with hepatic lipid synthesis, mitochondrial function, and immune regulation in a context-dependent manner [37–39].

#### Clinical and mechanistic implications

Clinically, these findings suggest that individuals with similar BMI may differ in metabolic risk depending on their vitamin D status. Recognizing vitamin D deficiency as a metabolic modifier opens new avenues for risk stratification and intervention, especially in populations vulnerable to both conditions. However, clinical studies suggest that vitamin D supplementation may not elicit uniform benefits, as overweight children have shown reduced responsiveness to replacement compared with normal-weight peers [40]. This highlights the need for tailored supplementation strategies in pediatric obesity. Prior studies have also shown that vitamin D signaling influences lipid metabolism through effects on hepatic lipid synthesis, lipoprotein remodeling enzymes such as lipoprotein lipase (LPL) and cholesteryl ester transfer protein (CETP), and inflammatory pathways that contribute to insulin resistance [11, 41, 42]. These mechanistic links provide biological plausibility for the modifying role of vitamin D in obesity-related metabolic profiles observed here.

From a pediatric perspective, the metabolomic signatures identified here may help refine early screening approaches by identifying children at elevated cardiometabolic risk despite similar BMI classifications. The enrichment of VLDL-related markers and depletion of HDL-related metabolites, together with reduced protective amino acids, could serve as biomarkers to stratify metabolic health beyond conventional anthropometric measures. Likewise, assessment of vitamin D status in children with obesity may inform personalized management, as deficiency could indicate heightened vulnerability to adverse lipid remodeling and inflammatory responses. Integrating metabolomic profiling with vitamin D screening could therefore guide targeted nutritional supplementation, lifestyle interventions, or closer cardiometabolic monitoring in high-risk pediatric subgroups.

A subset of metabolite ratios further illustrates how vitamin D status may reshape obesity-related metabolic patterns. Six ratios, VLDL_TG_by_M_HDL_C, TG_by_PG_by_M_HDL_C, S_HDL_TG_by_M_HDL_C, M_LDL_P_by_S_LDL_C_pct, S_LDL_CE_by_S_LDL_C_pct, and S_LDL_P_by_S_LDL_C_pct, exhibited both negative interaction effects and negative associations with vitamin D deficiency. These ratios fall into two clusters: those involving triglyceride enrichment relative to HDL components (VLDL_TG_by_M_HDL_C, TG_by_PG_by_M_HDL_C, S_HDL_TG_by_M_HDL_C) and those capturing structural composition within small LDL subclasses (M_LDL_P_by_S_LDL_C_pct, S_LDL_CE_by_S_LDL_C_pct, S_LDL_P_by_S_LDL_C_pct). While none were associated with obesity alone, the significant interaction terms indicate that the relationship between obesity and these lipid features depends on vitamin D status. Specifically, vitamin D deficiency appears to dampen or alter how these markers respond to adiposity. This pattern may reflect impaired lipid remodeling or reduced responsiveness of lipoprotein pathways under conditions of vitamin D deficiency.

## Conclusion

Our study reveals that obesity induces widespread and coordinated alterations in the metabolome, encompassing amino acid metabolism, lipid transport, lipoprotein remodeling, and inflammatory activation. These findings extend beyond established markers to highlight the compositional and functional remodeling of lipoproteins, particularly the depletion of large, cholesterol-rich HDL particles and the systemic upregulation of triglyceride-rich VLDL subclasses as central features of the obesity-associated metabolic phenotype. We also observed a signal suggesting that vitamin D deficiency may modify the metabolic impact of obesity. Although no individual metabolite interactions met stringent significance thresholds, the global enrichment of nominally significant interaction effects raises the possibility of a distributed, context-sensitive modulation of obesity-related pathways by vitamin D status. These findings should be interpreted as exploratory and require confirmation in larger or longitudinal studies.

Several limitations warrant consideration. First, the cross-sectional design precludes causal inference and restricts our ability to determine the temporal sequence of observed associations. Second, because this study was conducted in a clinical setting, detailed lifestyle and environmental data, including diet, socioeconomic status, physical activity, and sunlight exposure, were not systematically collected. The absence of these potential confounders may limit the interpretation and generalizability of our findings. Third, the modest sample size limited statistical power to detect small interaction effects, particularly after correction for multiple testing. Future research should build on these findings through prospective cohort studies, intervention trials such as vitamin D supplementation, and integrative multi-omics approaches to clarify causal pathways and identify modifiable metabolic targets. Such efforts will be essential for improving risk prediction and developing precision strategies for obesity-related disorders.

## Supplementary information

Supplementary Materials

Supplementary Table 1,2,3

Supplementary File S1

Supplementary File S2

Supplementary File S3

## Data Availability

The primary data generated in this study are available through the International HundredK+ Cohorts Consortium (IHCC). Additional data can be requested from the corresponding author, HH, by reasonable request.

## Code availability

The R code used to perform pediatric BMI classification is available at: https://github.com/huiqi-qu/pediatric-bmi-classification.
