# Supplementary material for: Metabolic remodeling and the modulatory role of vitamin D deficiency in African American children and adolescents with obesity
Source: Int J Obes (Lond). 2026 Jan 12;50(4):777–87. doi: 10.1038/s41366-025-02003-0 (PMC13056574; doi:10.1038/s41366-025-02003-0)
Supplement: Supplementary file 1 — Supplementary Materials [file 41366_2025_2003_MOESM1_ESM.docx]

**Metabolic Remodeling and the Modulatory Role of Vitamin D Deficiency in African American Children and Adolescents with Obesity**

Hui-Qi Qu, John J Connolly, Frank Mentch, Joseph Glessner, Hakon Hakonarson

**Supplementary Materials**

**Supplementary Table 1** Correlation of individual metabolite markers and obesity with age, sex, and vitamin D deficiency as covariates

**Supplementary Table 2** Correlation of individual metabolite markers and vitamin D deficiency with age, sex, and obesity as covariates

**Supplementary Table 3** Interaction effects between obesity and vitamin D deficiency on metabolite levels, with age and sex as covariates

**Supplementary File S1. Phenotype-ordered heatmaps by metabolite class.** One heatmap is shown for each metabolite class (e.g., amino acids, fatty acids, lipoproteins and subclasses, apolipoproteins, cholesteryl esters, free cholesterol, phospholipids, triglycerides, glycolysis-related metabolites, ketone bodies, fluid balance, total and relative lipoprotein lipids, particle concentrations, and particle sizes). For each class, cell values represent z-scored marker intensities across all individuals. Rows (samples) are first grouped by obesity status (without obesity, then with obesity) and, within each group, hierarchically ordered using correlation distance (1 − Spearman’s ρ) with Ward’s linkage. Marker columns are clustered within each class using the same linkage on correlation distance. Row annotations indicate obesity status and vitamin D deficiency. The color scale represents relative abundance (standardized units).

**Supplementary File S2. Spearman correlation heatmaps by metabolite class.** One heatmap is shown for each metabolite class (e.g., amino acids, fatty acids, apolipoproteins, cholesterol and cholesteryl esters, free cholesterol, phospholipids, triglycerides, glycolysis-related metabolites, ketone bodies, fluid balance, other lipid species, total and relative lipoprotein lipids, particle concentrations, particle sizes, and lipoprotein subclasses). Each heatmap displays the pairwise Spearman correlation coefficients (ρ) between markers within the given class across all individuals. Hierarchical clustering was applied using Ward’s method on the distance metric (1 − |ρ|), grouping metabolites with similar correlation patterns. Red indicates positive correlations, and blue indicates negative correlations.

**Supplementary File S3. Principal component analysis (PCA) of the plasma metabolome by age.** PCA was performed on normalized NMR-based metabolomic profiles from African American children and adolescents. (Page 1) Scree plot showing the proportion of total variance explained by each principal component (PC). PC1 and PC2 accounted for 34.5% and 21.5% of total variance, respectively, with the first four PCs cumulatively explaining 73.8% of total variability. The red line indicates cumulative variance explained. (Page 2) PCA score plot of PC1 vs. PC2 colored by chronological age (years) and shaped by obesity status (without obesity vs. with obesity). (Page 3) PCA score plot of PC1 vs. PC2 colored by pediatric age group (2–5, 6–11, 12–15, and 16–21 years) and shaped by obesity status. Substantial overlap across age strata indicates limited subclass clustering by developmental stage, suggesting that obesity was the dominant driver of metabolomic variation in this cohort. No clear gradient or clustering by age was observed, indicating that age contributed modestly to global metabolic variance after accounting for obesity.
