# Supplementary material for: Metabolic remodeling and the modulatory role of vitamin D deficiency in African American children and adolescents with obesity
Source: Int J Obes (Lond). 2026 Jan 12;50(4):777–87. doi: 10.1038/s41366-025-02003-0 (PMC13056574; doi:10.1038/s41366-025-02003-0)

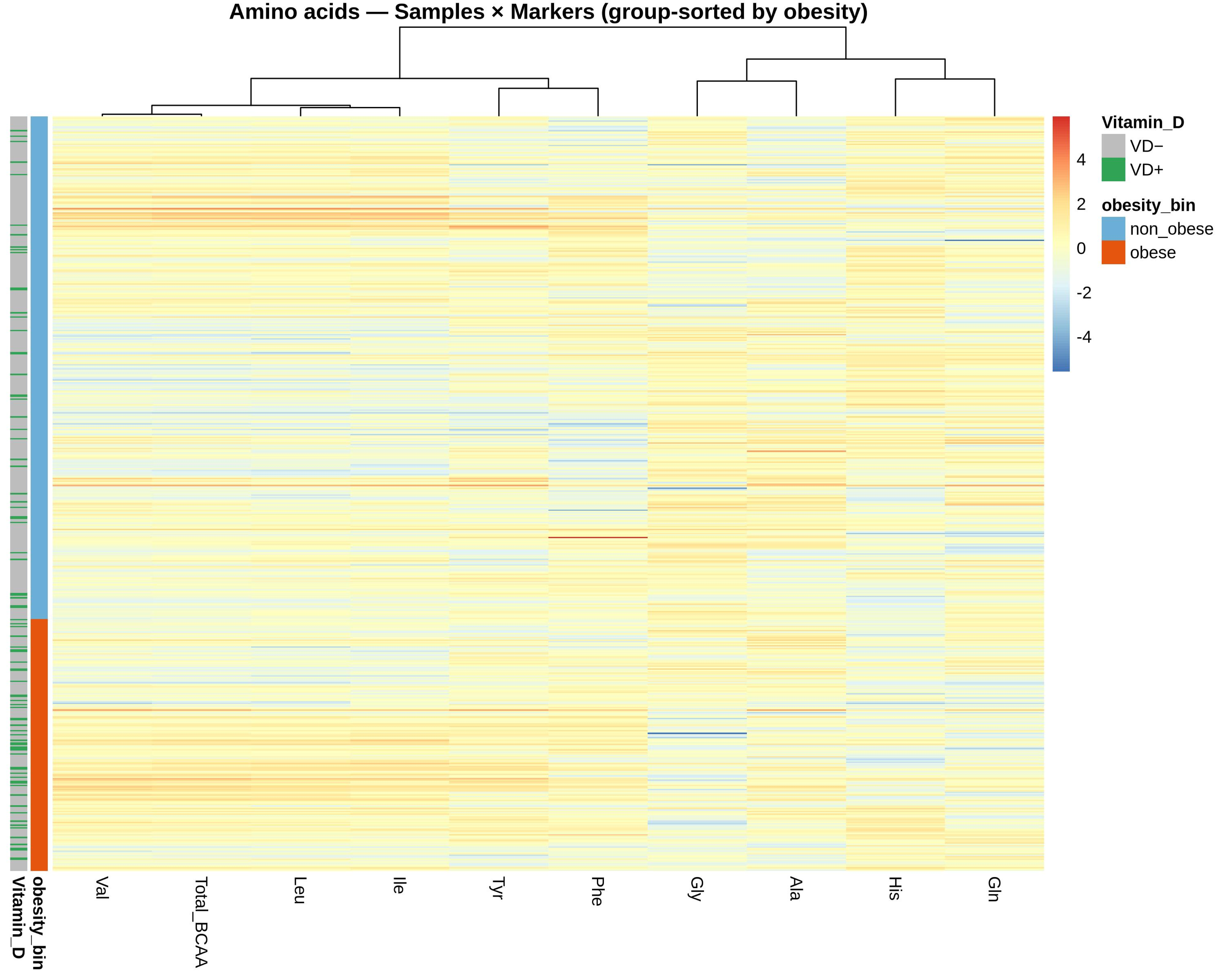
Amino acids — Samples × Markers (group-sorted by obesity)
Vitamin_D
VD−
VD+
obesity_bin
non_obese
obese
4
2
0
-2
-4
Val
Total_BCAA
Leu
Ile
Tyr
Phe
Gly
Ala
His
Gln
Vitamin_D
obesity_bin

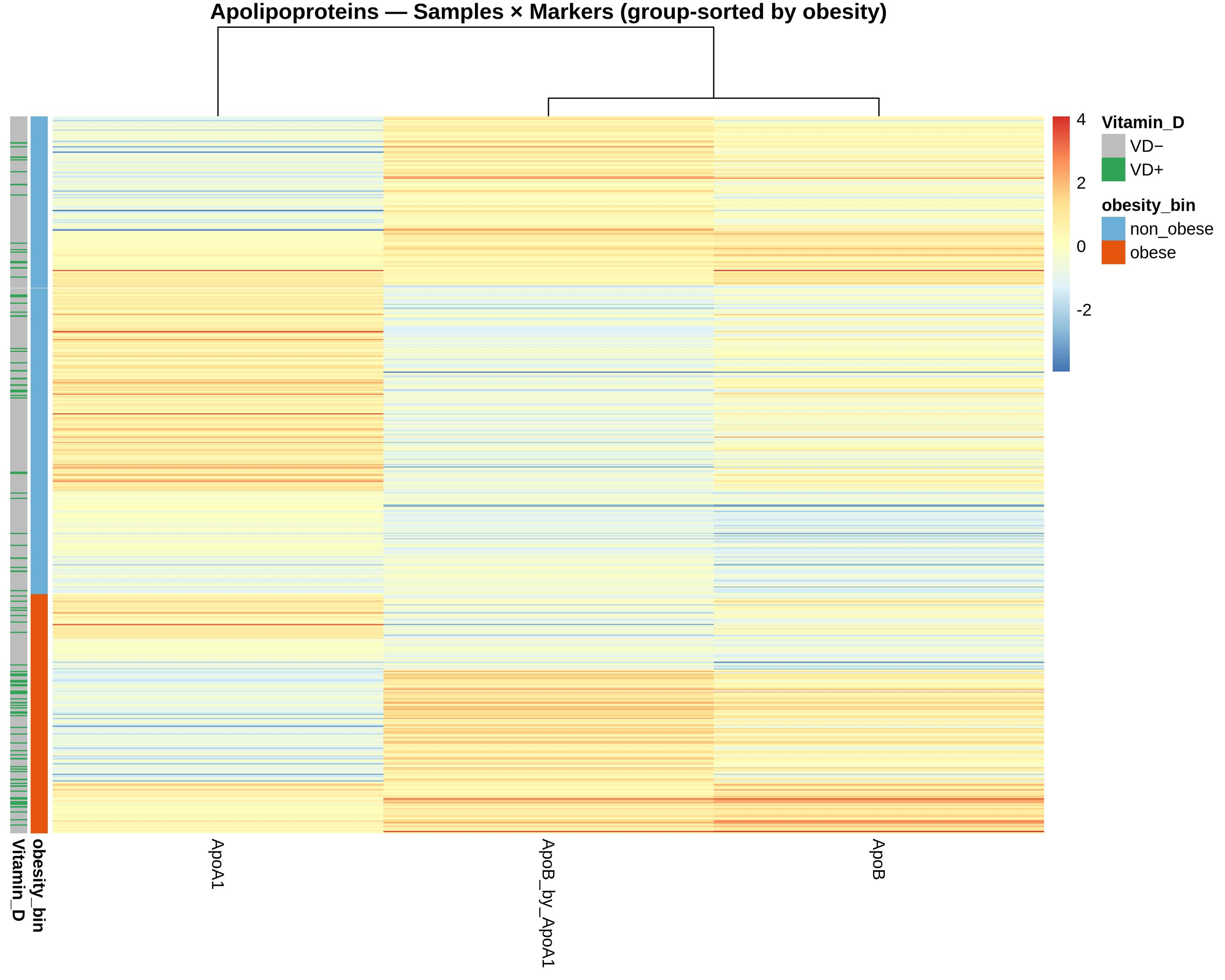
Apolipoproteins — Samples × Markers (group-sorted by obesity)
Vitamin_D
VD−
VD+
obesity_bin
non_obese
obese
4
2
0
-2
Vitamin_D
obesity_bin
ApoA1
ApoB_by_ApoA1
ApoB

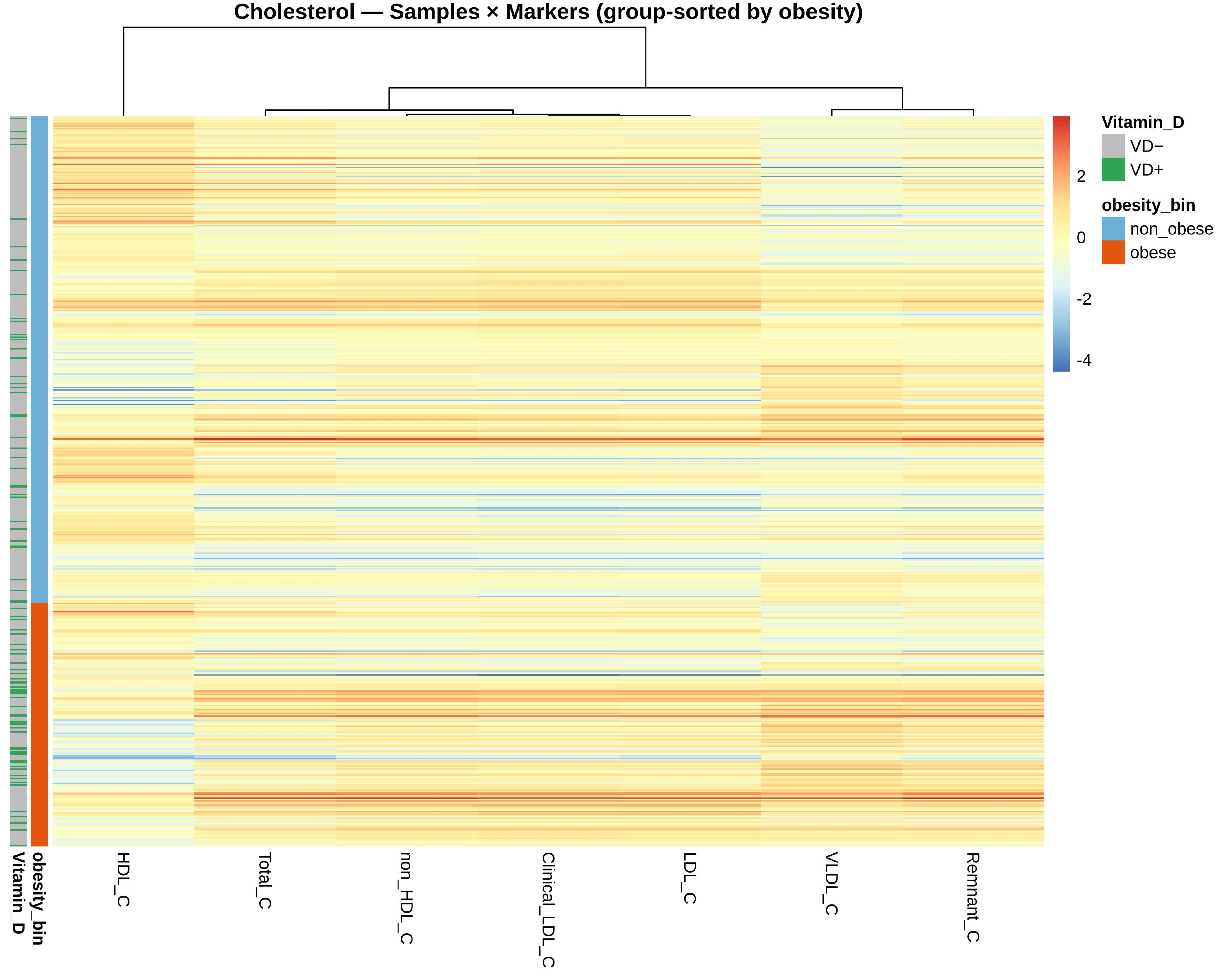
Cholesterol — Samples × Markers (group-sorted by obesity)
HDL_C
Total_C
non_HDL_C
Clinical_LDL_C
LDL_C
VLDL_C
Remnant_C
Vitamin_D
obesity_bin
2
0
-2
-4
Vitamin_D
VD−
VD+
obesity_bin
non_obese
obese

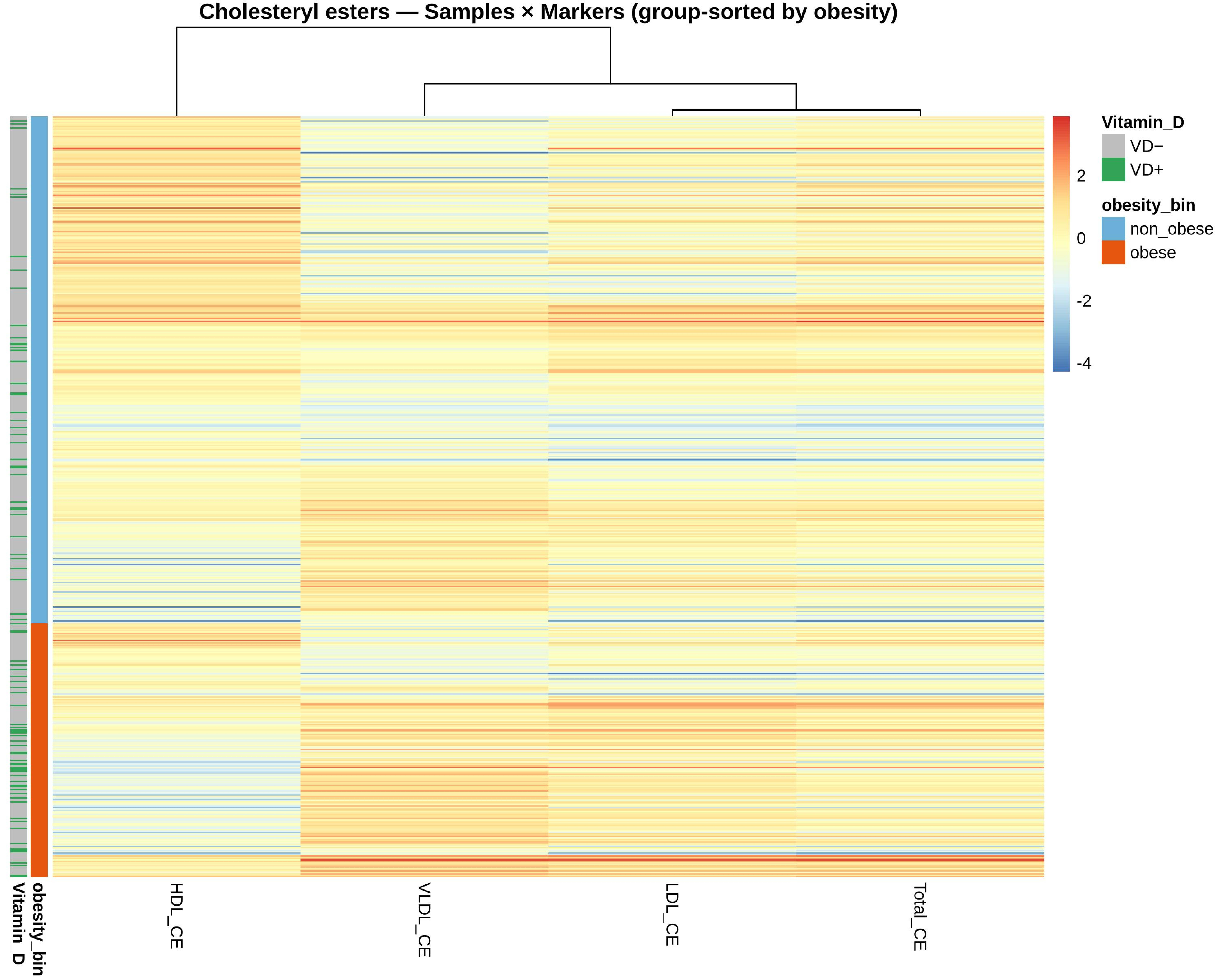

Cholesteryl esters — Samples × Markers (group-sorted by obesity)
Vitamin_D
VD−
VD+
obesity_bin
non_obese
obese
2
0
-2
-4
HDL_CE
VLDL_CE
LDL_CE
Total_CE
obesity_bin
Vitamin_D

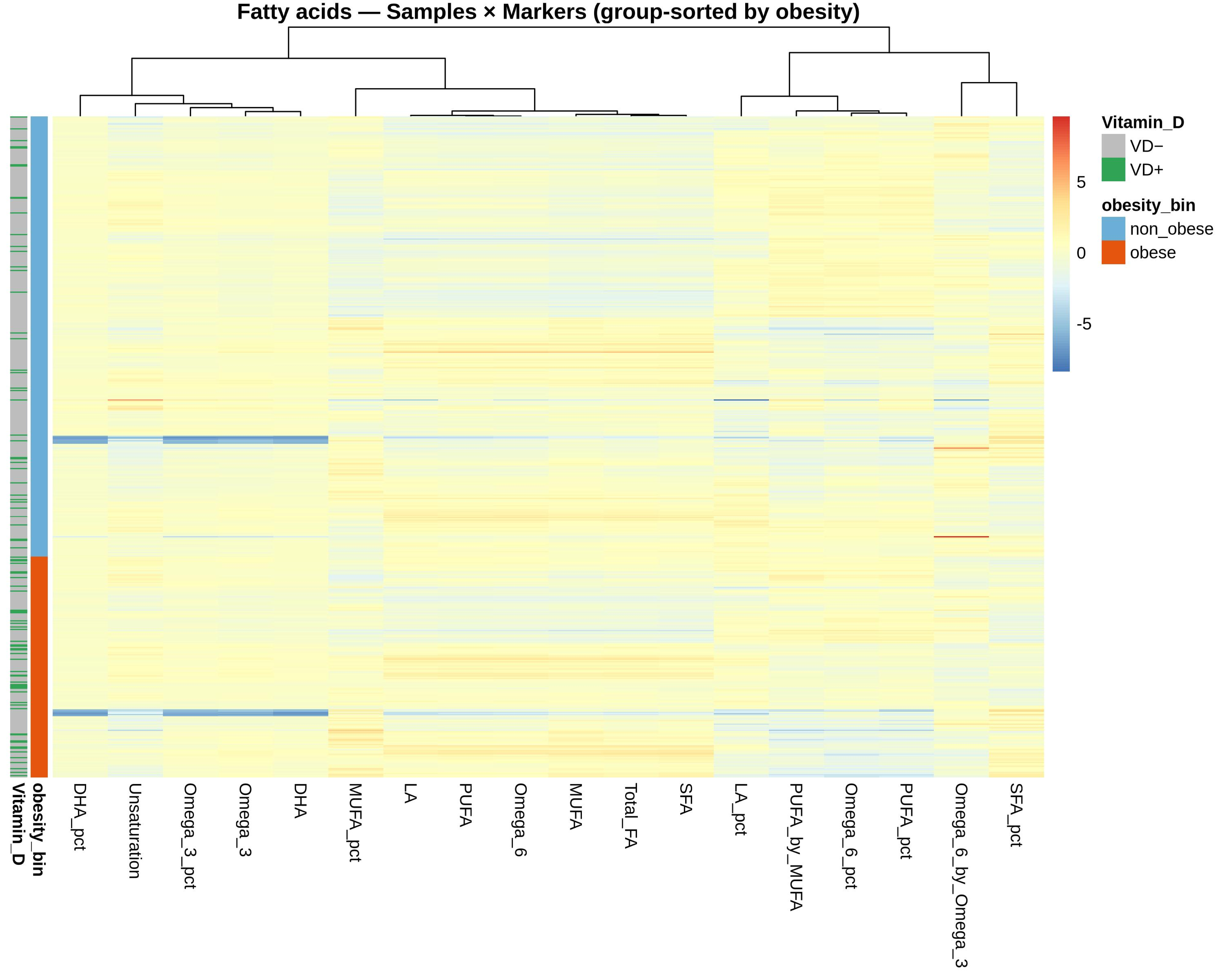

Fatty acids — Samples × Markers (group-sorted by obesity)
Vitamin_D
VD−
VD+
obesity_bin
non_obese
obese
5
0
-5
Vitamin_D
obesity_bin
DHA_pct
Unsaturation
Omega_3_pct
Omega_3
DHA
MUFA_pct
LA
PUFA
Omega_6
MUFA
Total_FA
SFA
LA_pct
PUFA_by_MUFA
Omega_6_pct
PUFA_pct
Omega_6_by_Omega_3
SFA_pct

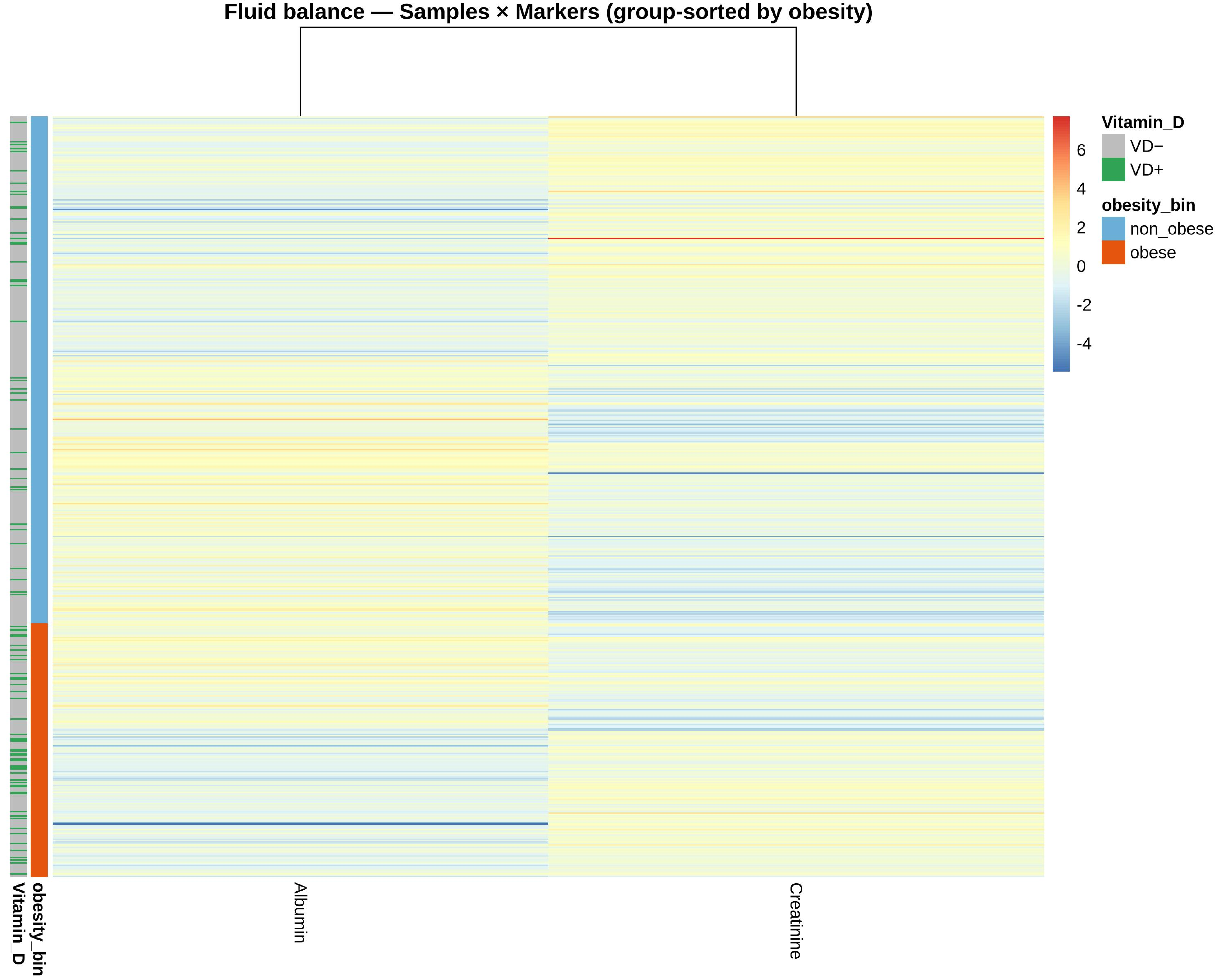
Fluid balance — Samples × Markers (group-sorted by obesity)
Vitamin_D
VD−
VD+
obesity_bin
non_obese
obese
6
4
2
0
-2
-4
Vitamin_D
obesity_bin
Albumin
Creatinine

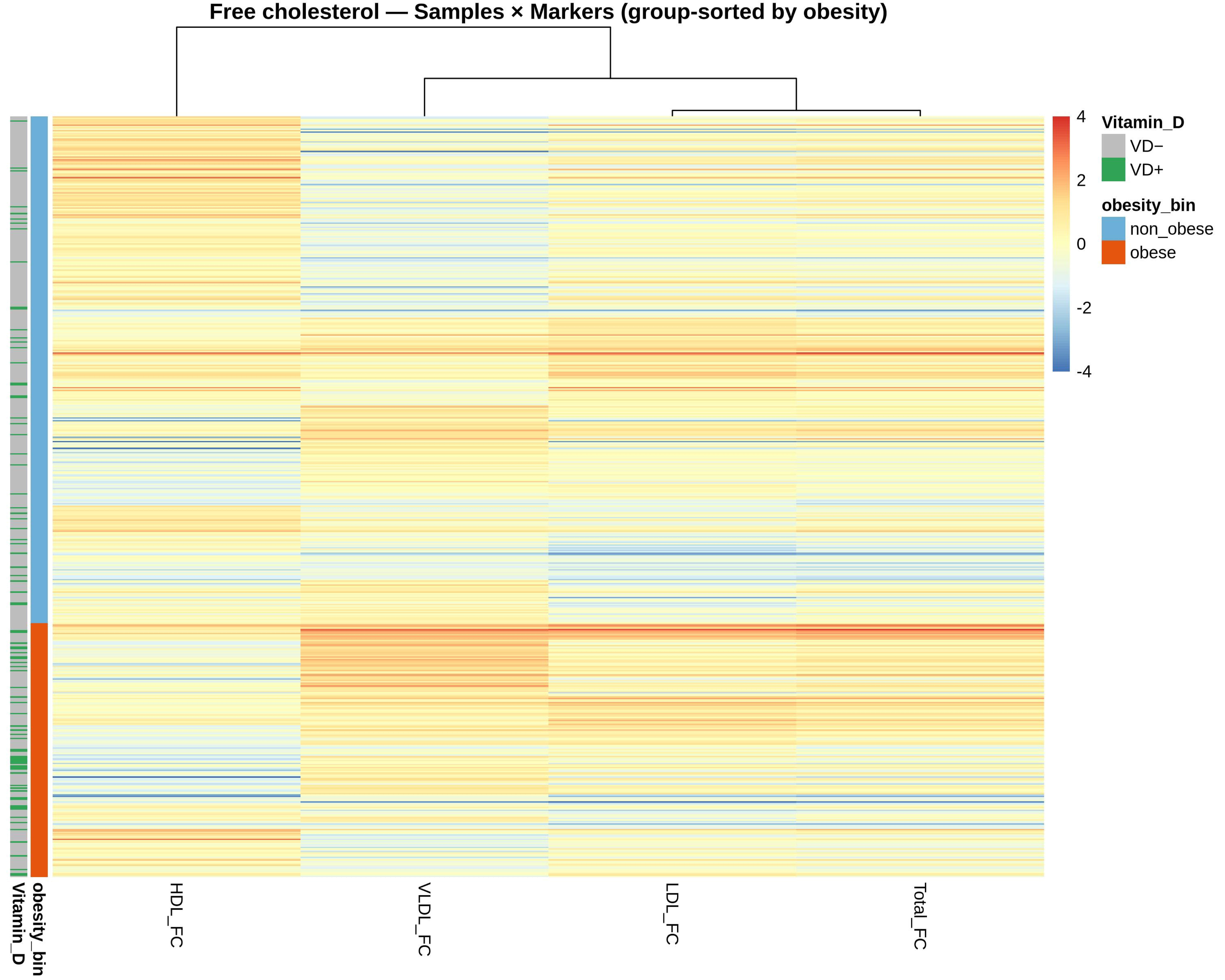
Free cholesterol — Samples × Markers (group-sorted by obesity)
4
2
0
-2
-4
Vitamin_D
VD−
VD+
obesity_bin
non_obese
obese
HDL_FC
VLDL_FC
LDL_FC
Total_FC
Vitamin_D
obesity_bin

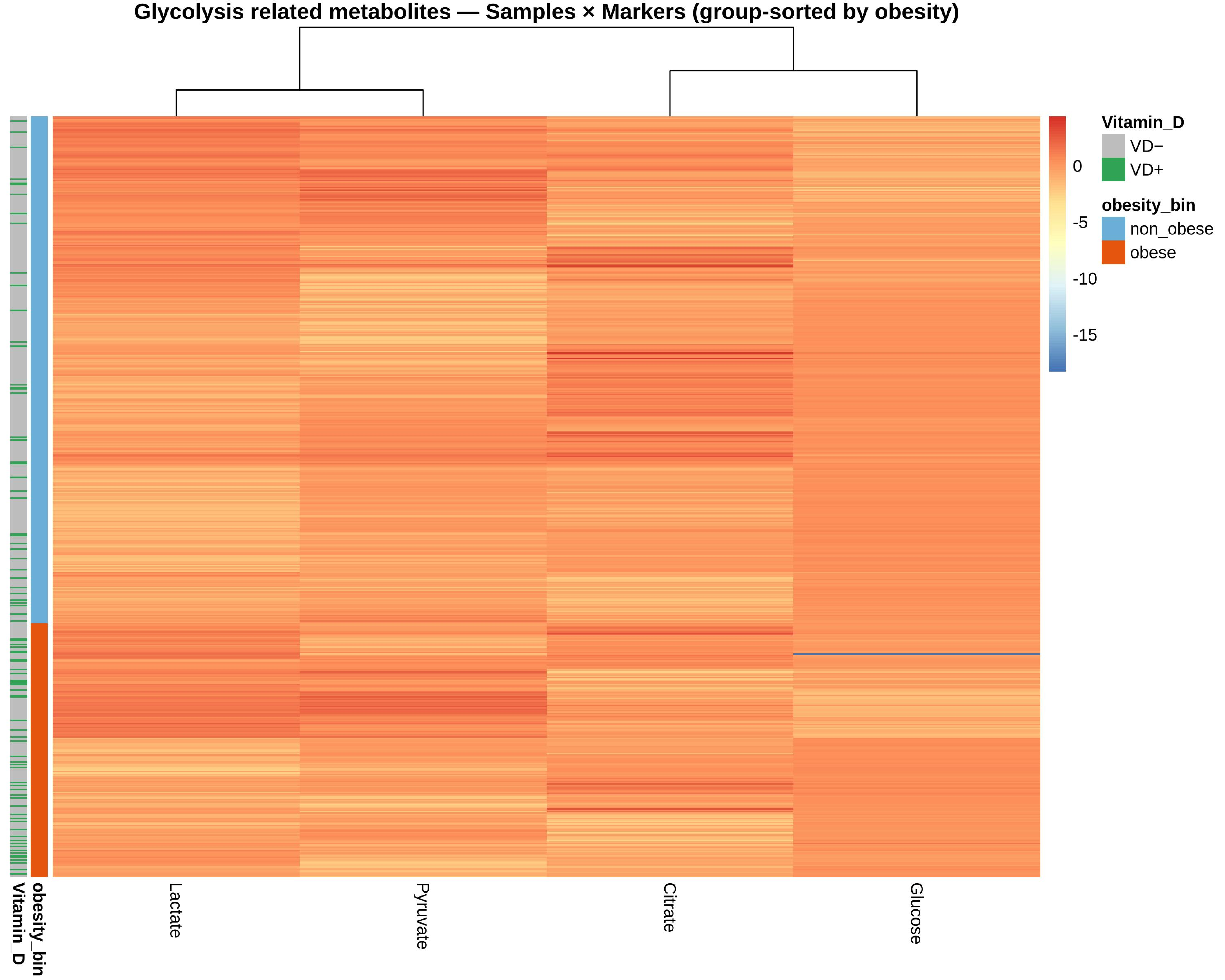

Glycolysis related metabolites — Samples × Markers (group-sorted by obesity)
Vitamin_D
VD−
VD+
obesity_bin
non_obese
obese
0
-5
-10
-15
Lactate
Pyruvate
Citrate
Glucose
obesity_bin
Vitamin_D

Ketone bodies — Samples × Markers (group-sorted by obesity)

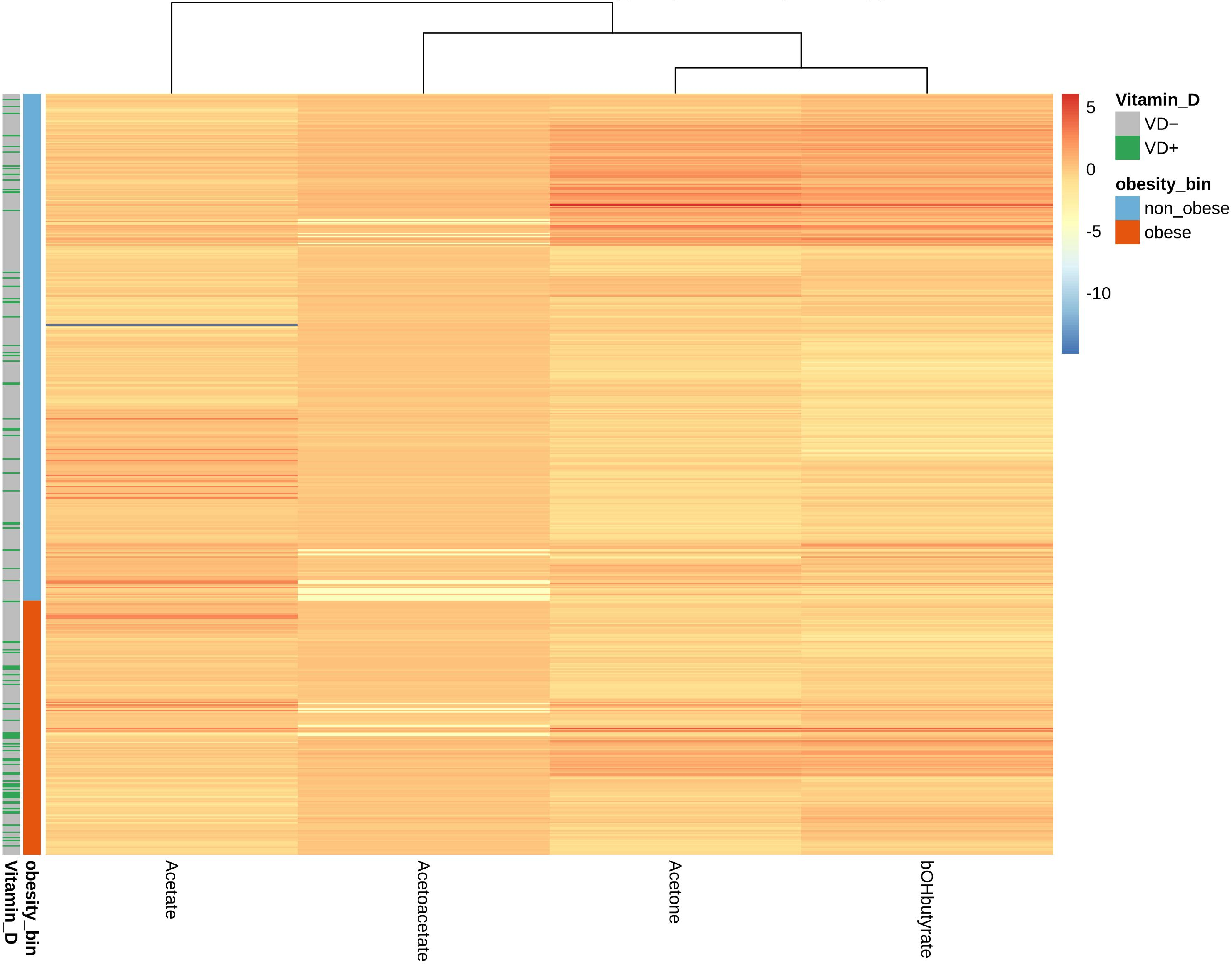

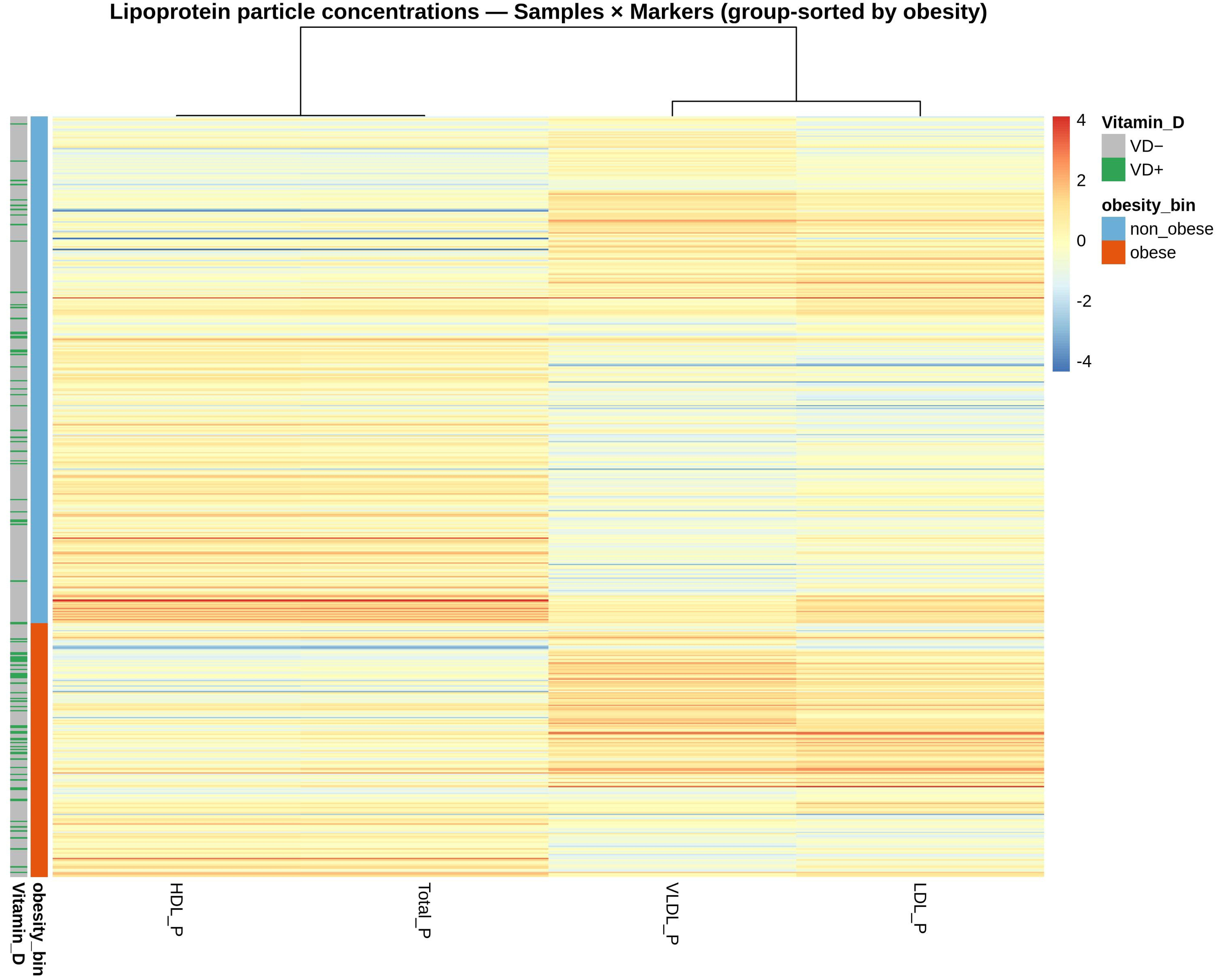

Lipoprotein particle concentrations — Samples × Markers (group-sorted by obesity)
Vitamin_D
VD−
VD+
obesity_bin
non_obese
obese
4
2
0
-2
-4
HDL_P
Total_P
VLDL_P
LDL_P
Vitamin_D
obesity_bin

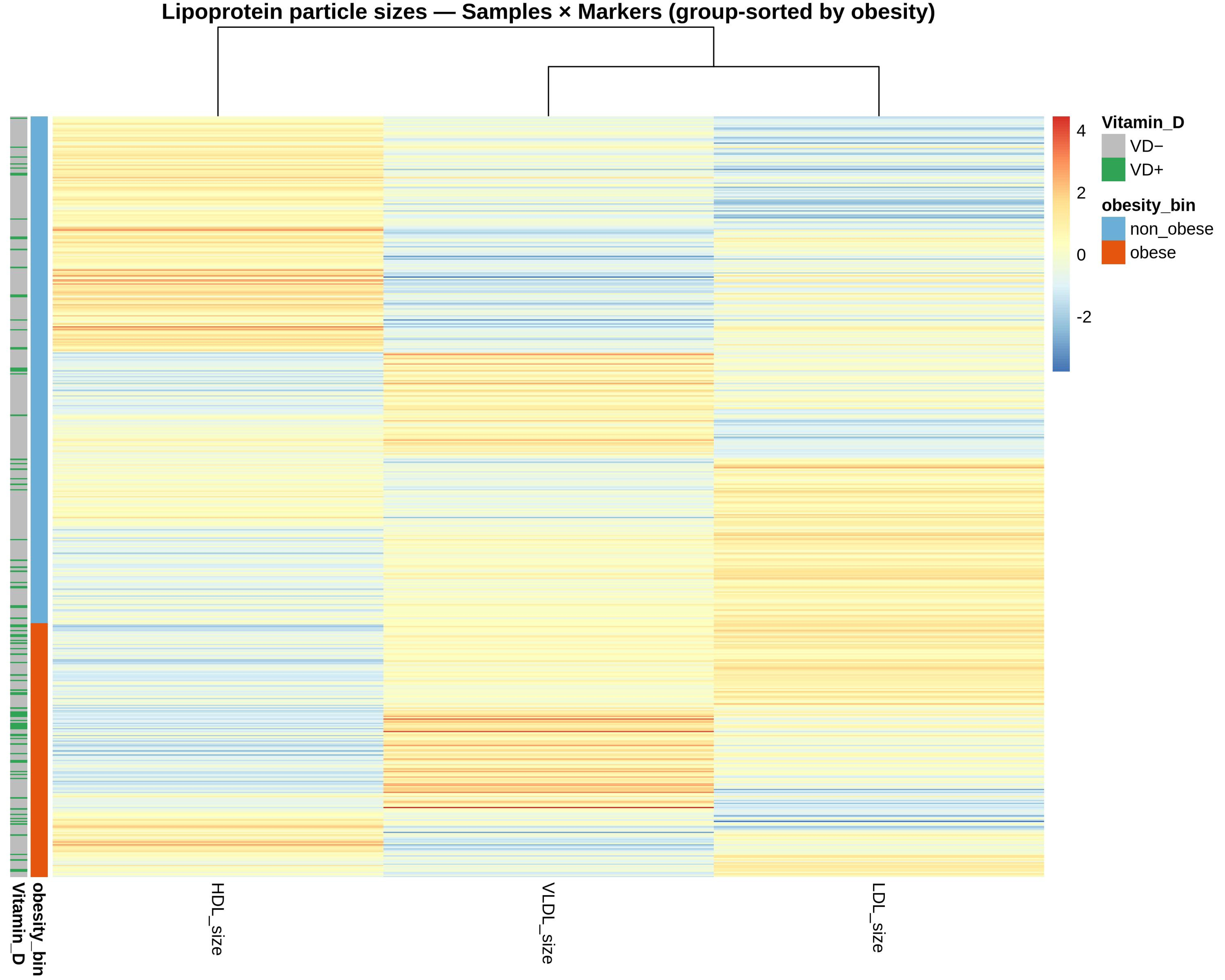
Lipoprotein particle sizes — Samples × Markers (group-sorted by obesity)
Vitamin_D
VD−
VD+
obesity_bin
non_obese
obese
4
2
0
-2
Vitamin_D
obesity_bin
HDL_size
VLDL_size
LDL_size

Lipoprotein subclasses — Samples × Markers (group-sorted by obesity)

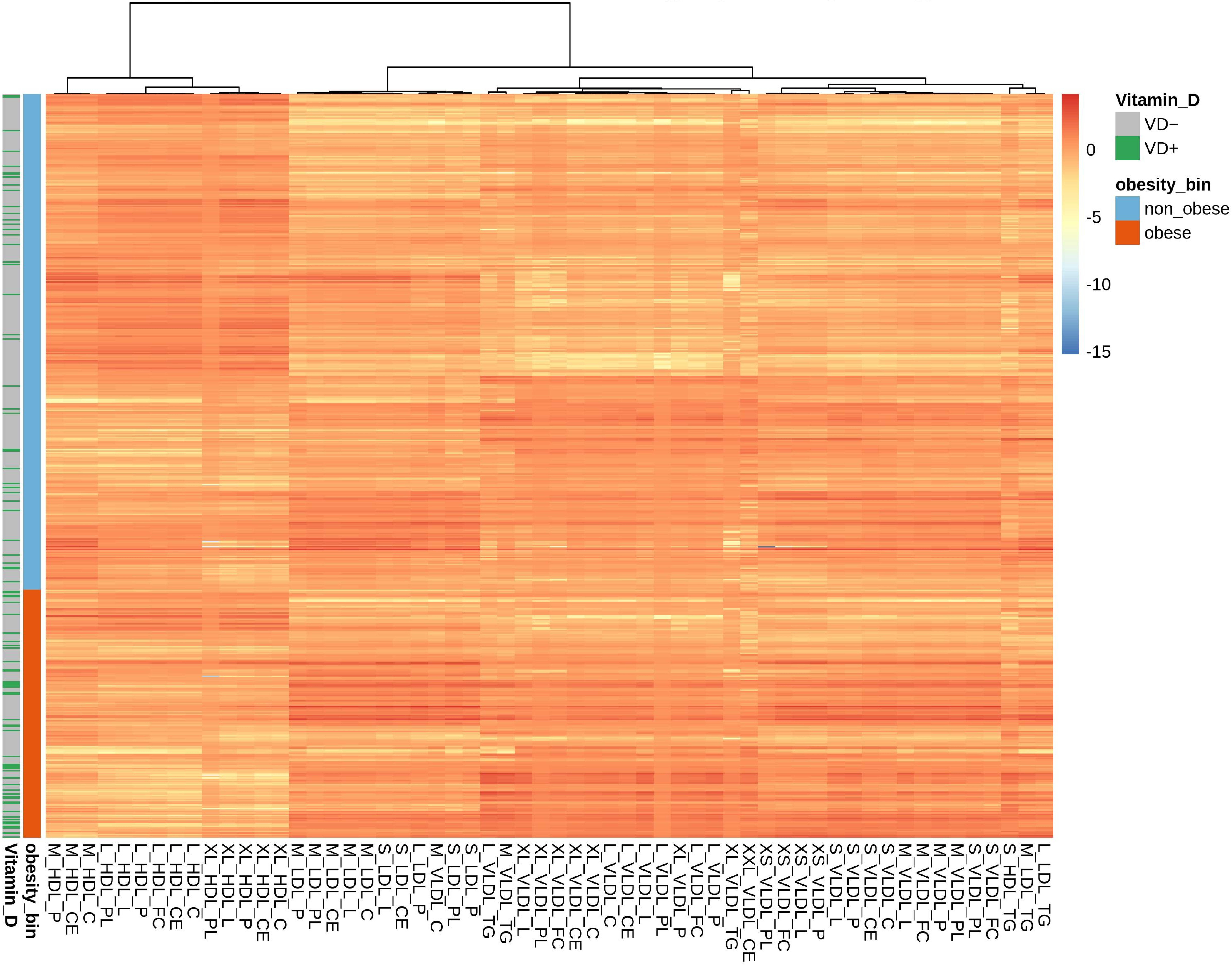

Other lipids — Samples × Markers (group-sorted by obesity)

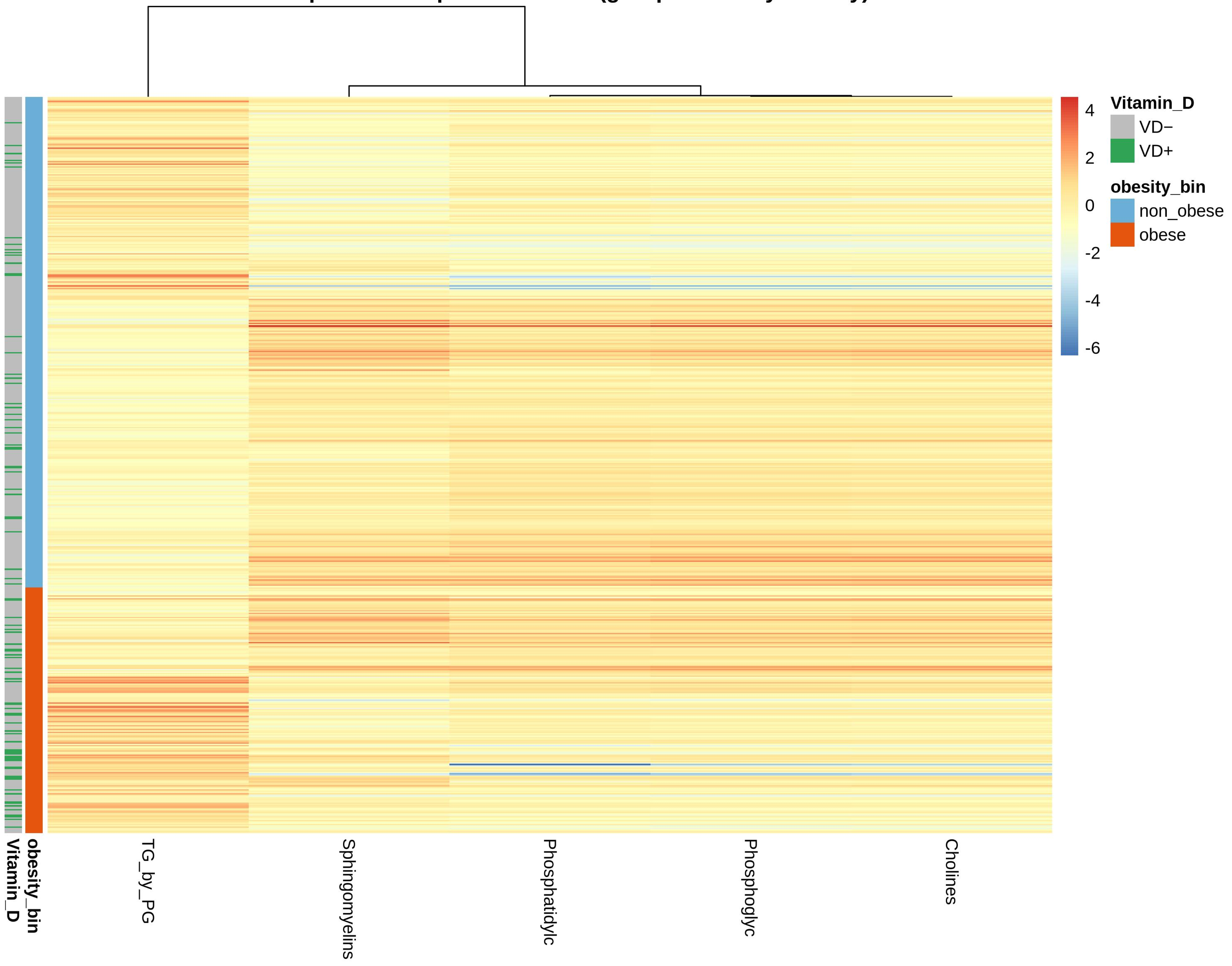

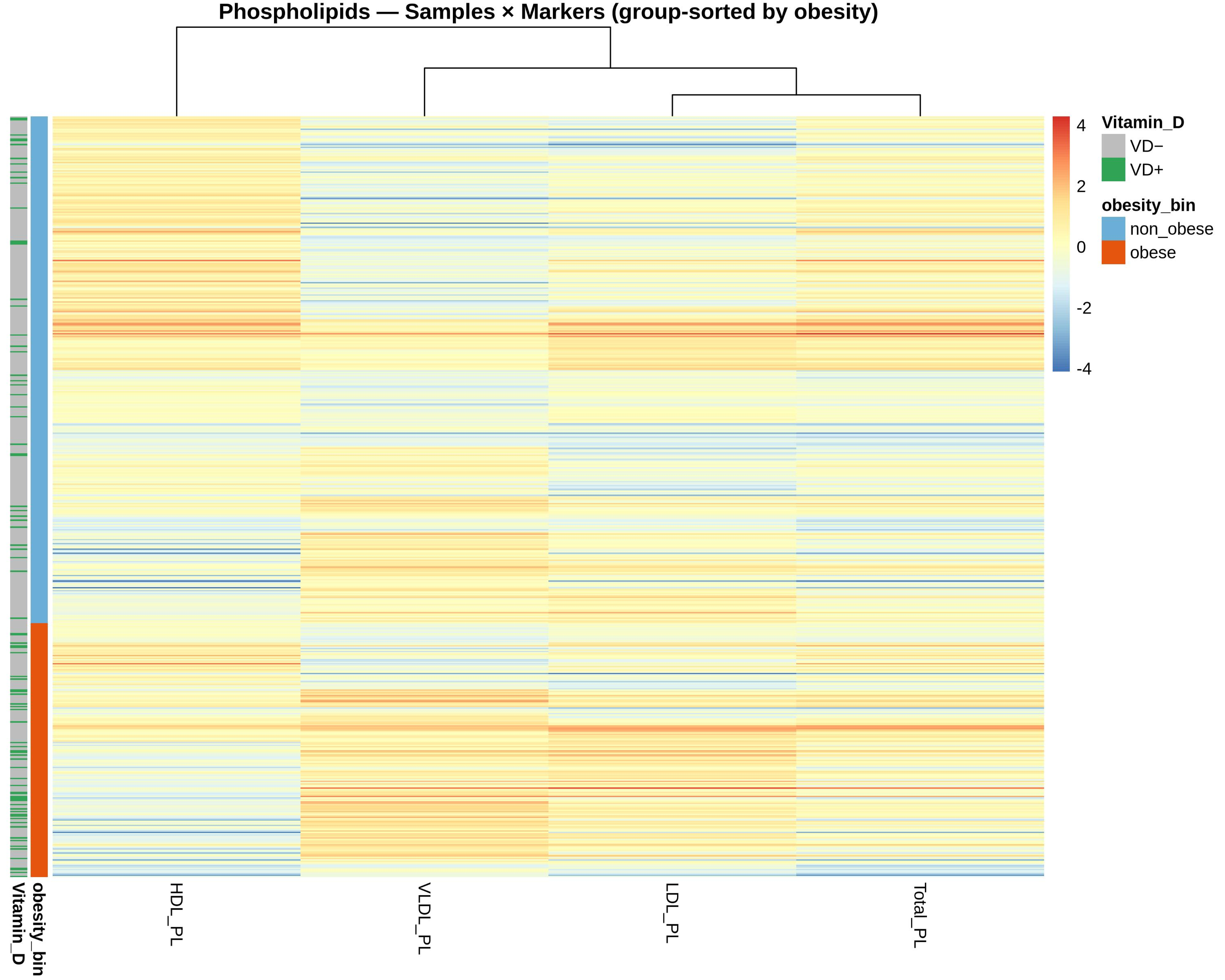

Phospholipids — Samples × Markers (group-sorted by obesity)
Vitamin_D
VD−
VD+
obesity_bin
non_obese
obese
4
2
0
-2
-4
HDL_PL
VLDL_PL
LDL_PL
Total_PL
obesity_bin
Vitamin_D

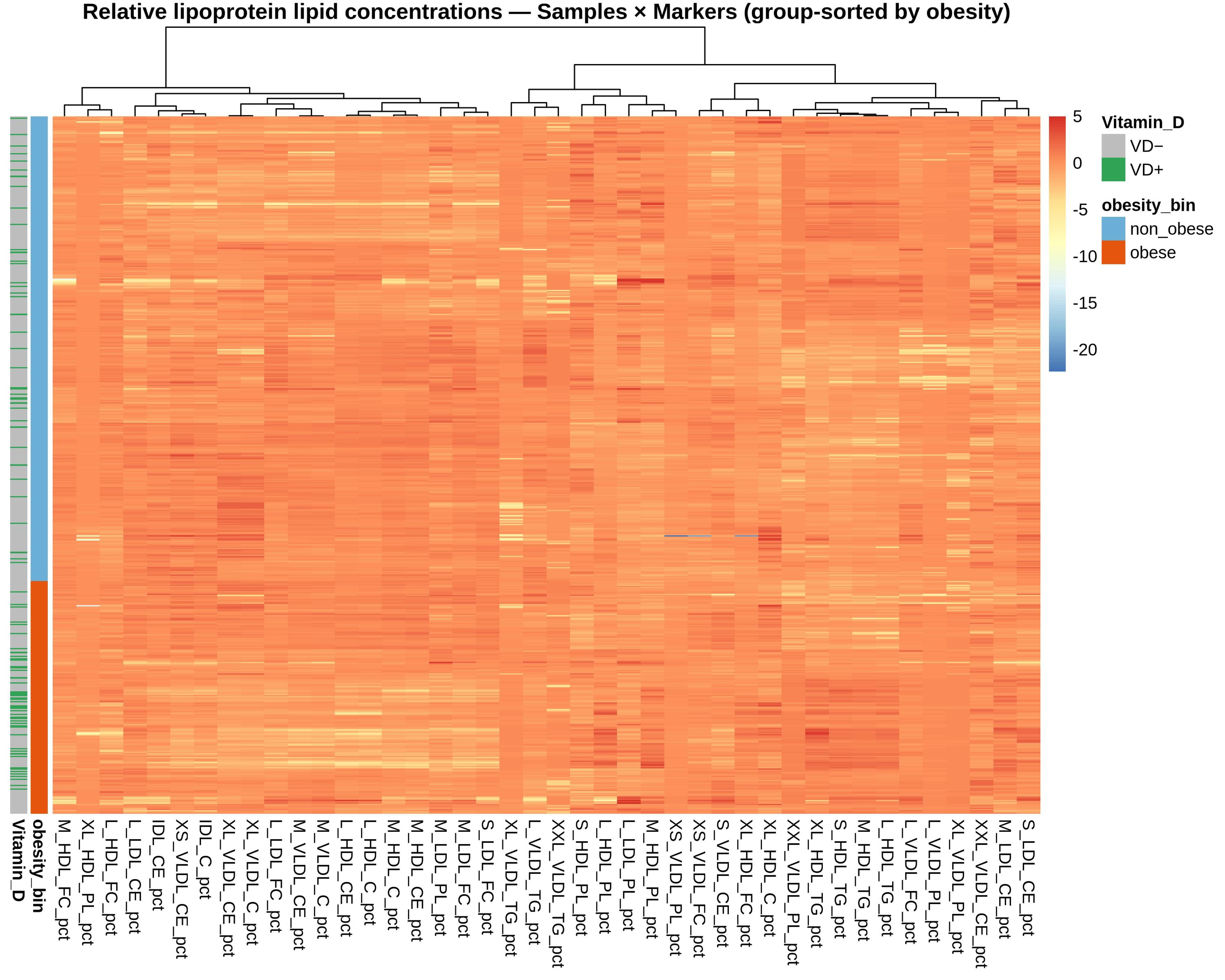
Relative lipoprotein lipid concentrations — Samples × Markers (group-sorted by obesity)
Vitamin_D
VD−
VD+
obesity_bin
non_obese
obese
5
0
-5
-10
-15
-20
Vitamin_D
obesity_bin
M_HDL_FC_pct
XL_HDL_PL_pct
L_HDL_FC_pct
L_LDL_CE_pct
IDL_CE_pct
XS_VLDL_CE_pct
IDL_C_pct
XL_VLDL_CE_pct
XL_VLDL_C_pct
L_LDL_FC_pct
M_VLDL_CE_pct
M_VLDL_C_pct
L_HDL_CE_pct
L_HDL_C_pct
M_HDL_C_pct
M_HDL_CE_pct
M_LDL_PL_pct
M_LDL_FC_pct
S_LDL_FC_pct
XL_VLDL_TG_pct
L_VLDL_TG_pct
XXL_VLDL_TG_pct
S_HDL_PL_pct
L_HDL_PL_pct
L_LDL_PL_pct
M_HDL_PL_pct
XS_VLDL_PL_pct
XS_VLDL_FC_pct
S_VLDL_CE_pct
XL_HDL_FC_pct
XL_HDL_C_pct
XXL_VLDL_PL_pct
XL_HDL_TG_pct
S_HDL_TG_pct
M_HDL_TG_pct
L_HDL_TG_pct
L_VLDL_FC_pct
L_VLDL_PL_pct
XL_VLDL_PL_pct
XXL_VLDL_CE_pct
M_LDL_CE_pct
S_LDL_CE_pct

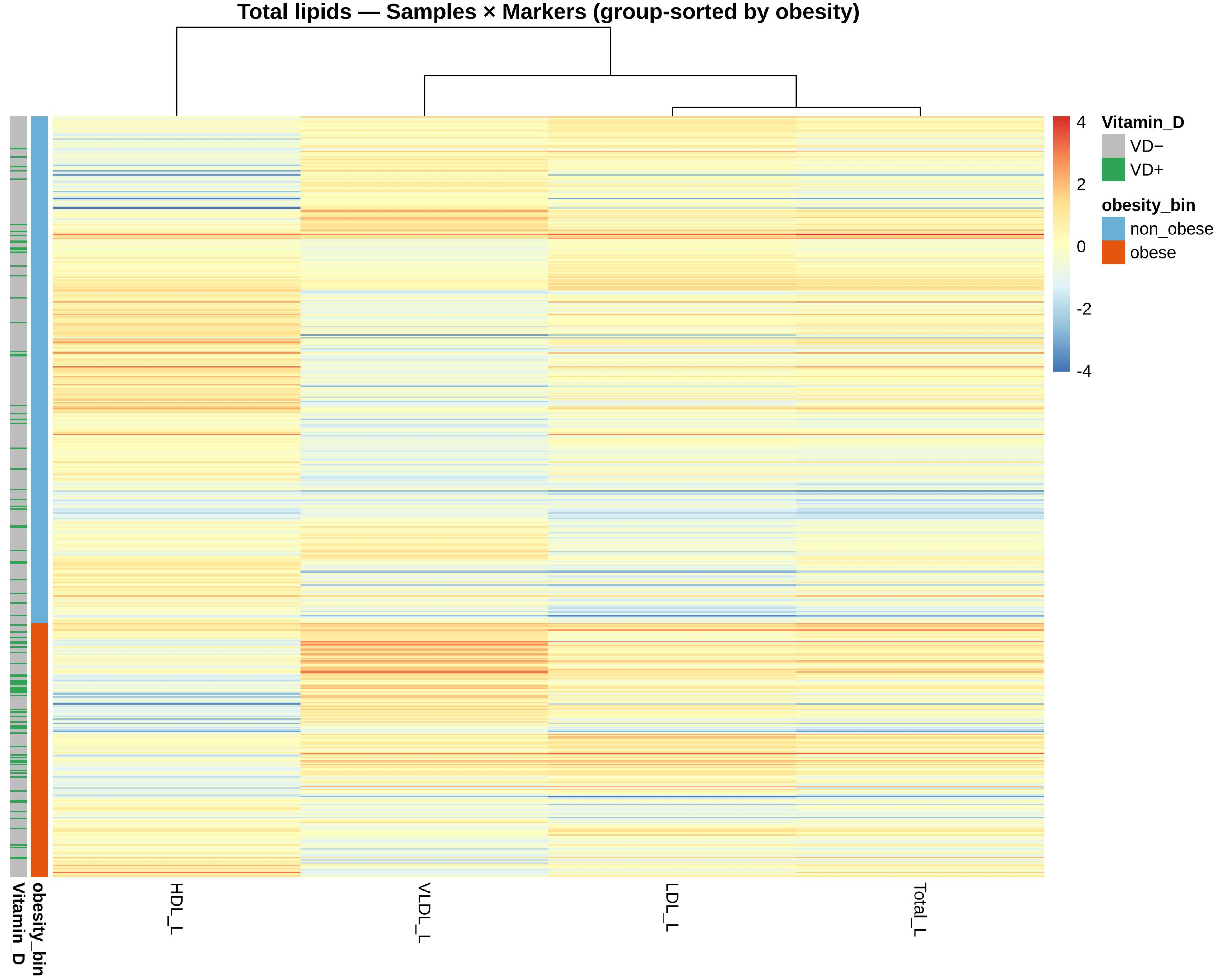
Total lipids — Samples × Markers (group-sorted by obesity)
HDL_L
VLDL_L
LDL_L
Total_L
Vitamin_D
obesity_bin
4
2
0
-2
-4
Vitamin_D
VD−
VD+
obesity_bin
non_obese
obese

Triglycerides — Samples × Markers (group-sorted by obesity)

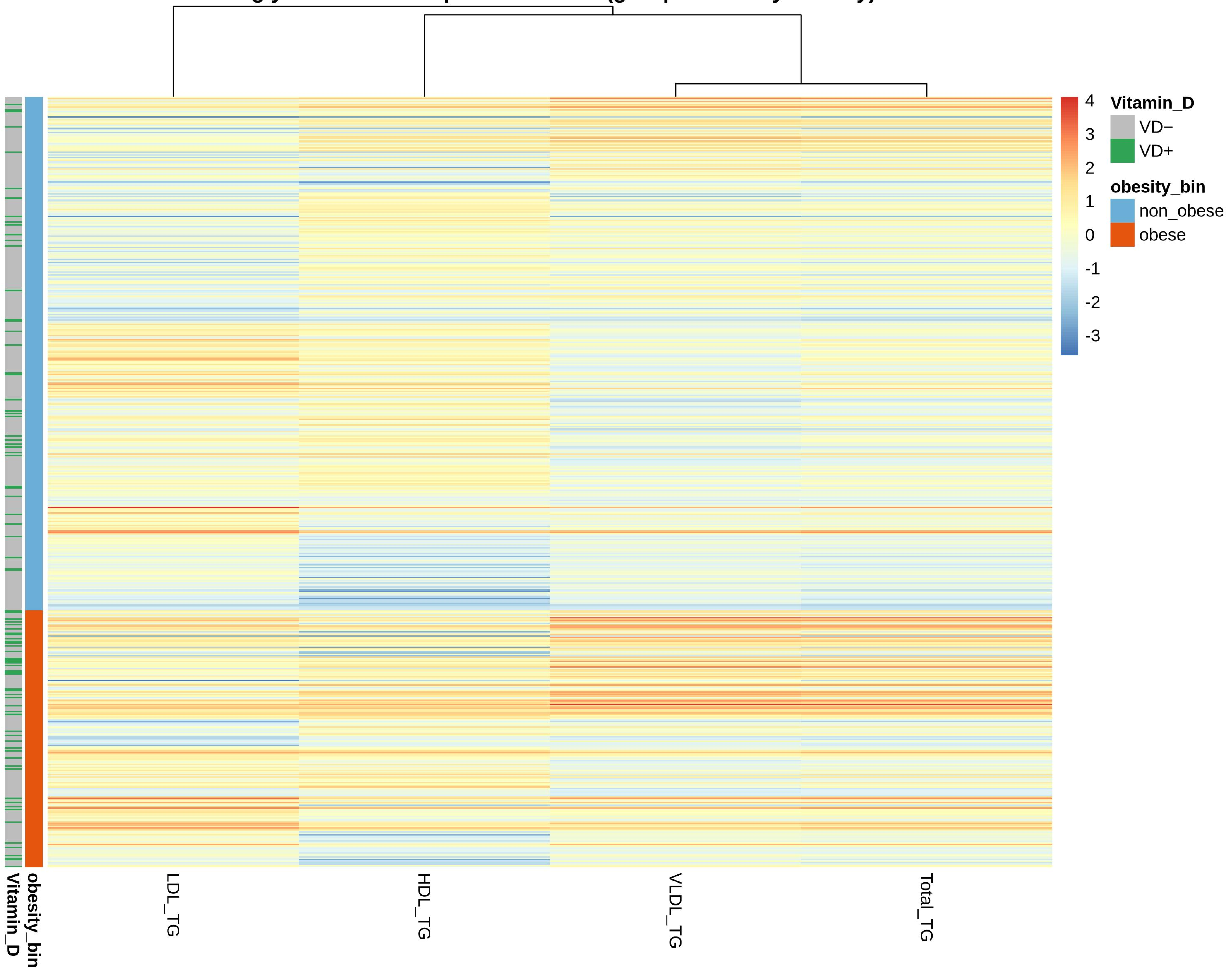

Supplement: Supplementary file 3 — Supplementary File S1 [file 41366_2025_2003_MOESM3_ESM.pdf]
