# Supplementary material for: Metabolic remodeling and the modulatory role of vitamin D deficiency in African American children and adolescents with obesity
Source: Int J Obes (Lond). 2026 Jan 12;50(4):777–87. doi: 10.1038/s41366-025-02003-0 (PMC13056574; doi:10.1038/s41366-025-02003-0)
Supplement: Supplementary file 5 — Supplementary File S3 [file 41366_2025_2003_MOESM5_ESM.pdf]

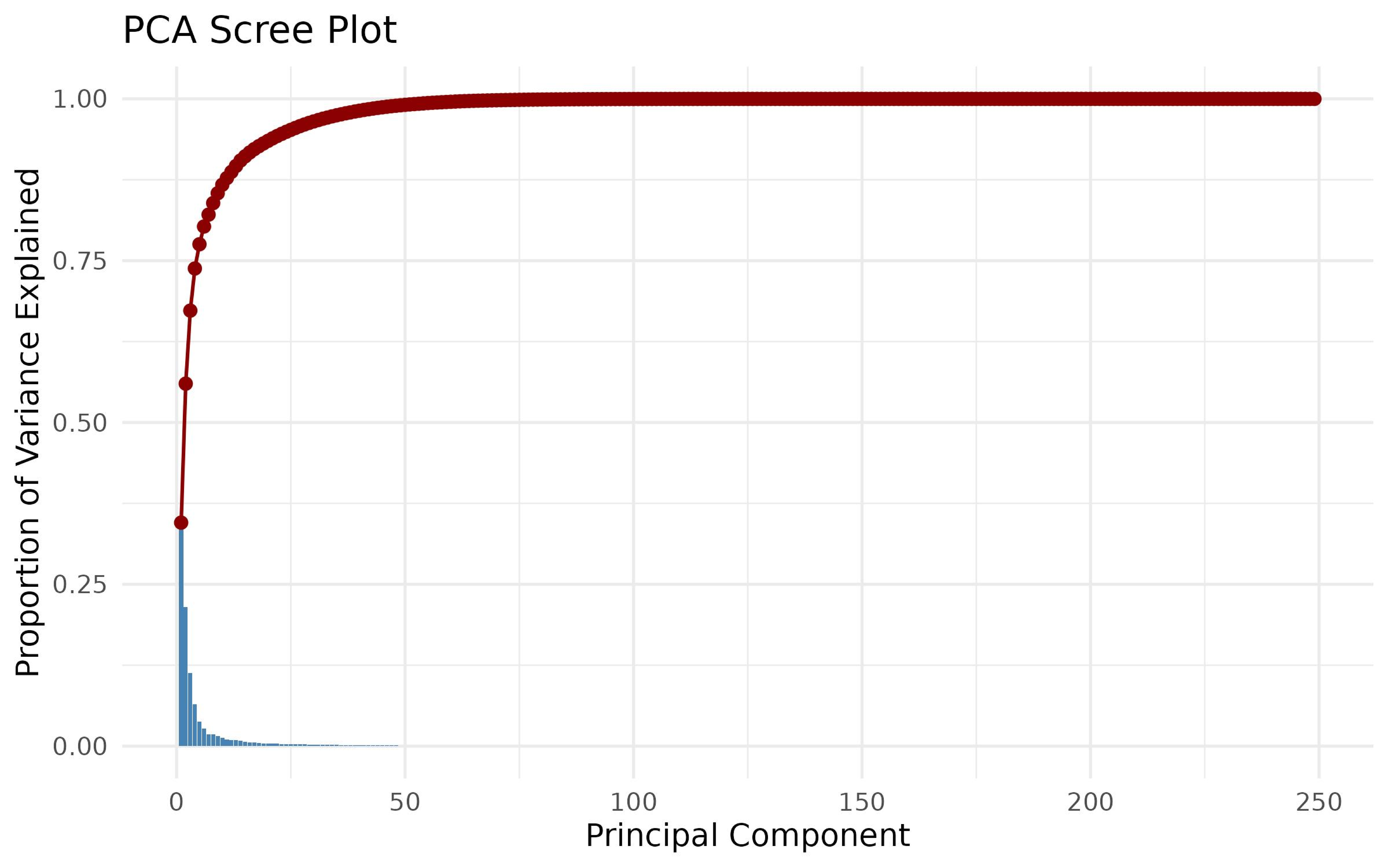
PCA Scree Plot
Proportion of Variance Explained
1.00
0.75
0.50
0.25
0.00
0
50
100
150
200
250
Principal Component

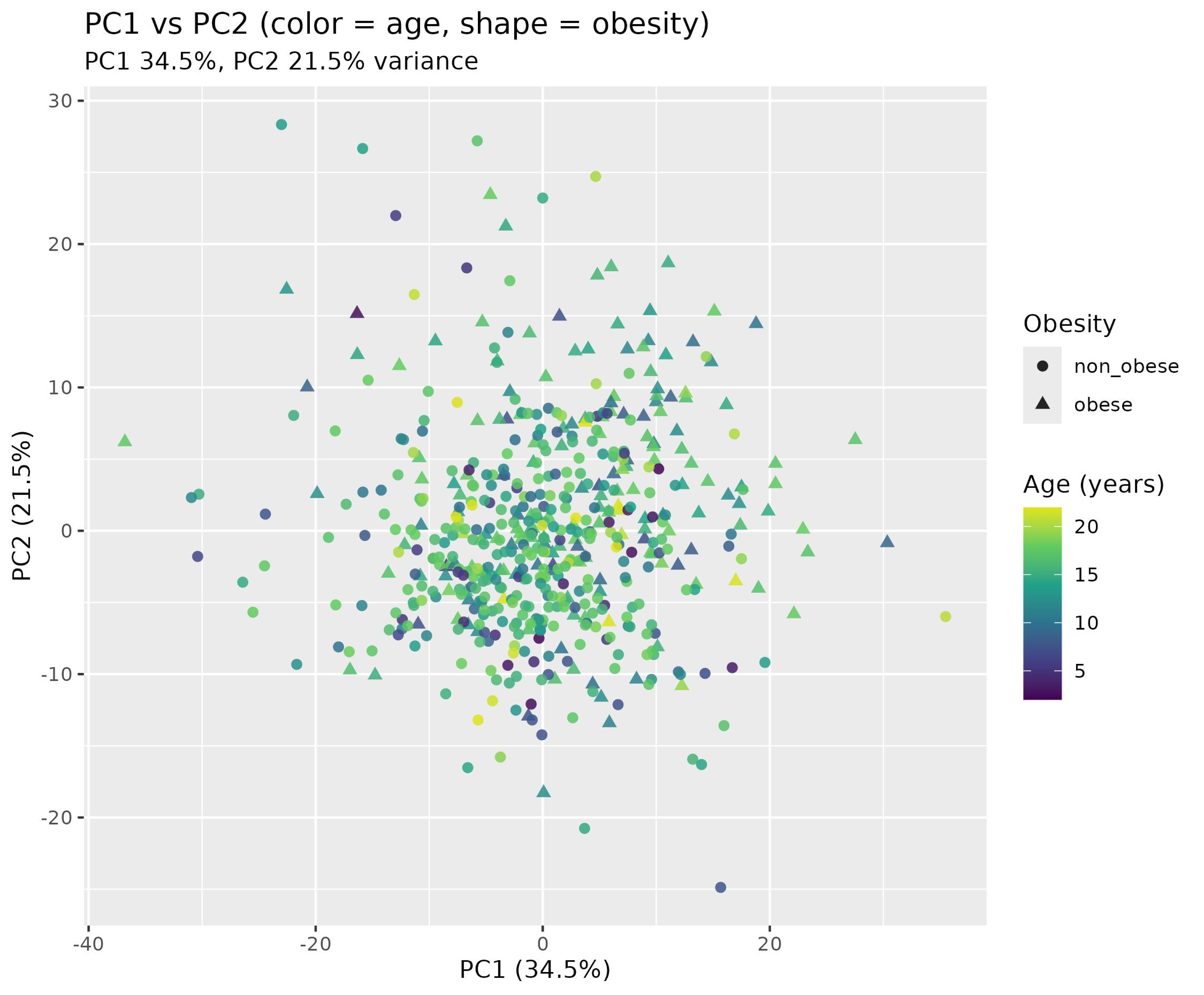

PC1 vs PC2 (color = age, shape = obesity)
PC1 34.5%, PC2 21.5% variance
PC2 (21.5%)
30
20
10
0
-10
-20
-40
-20
0
20
PC1 (34.5%)
Obesity
non_obese
obese
Age (years)
20
15
10
5

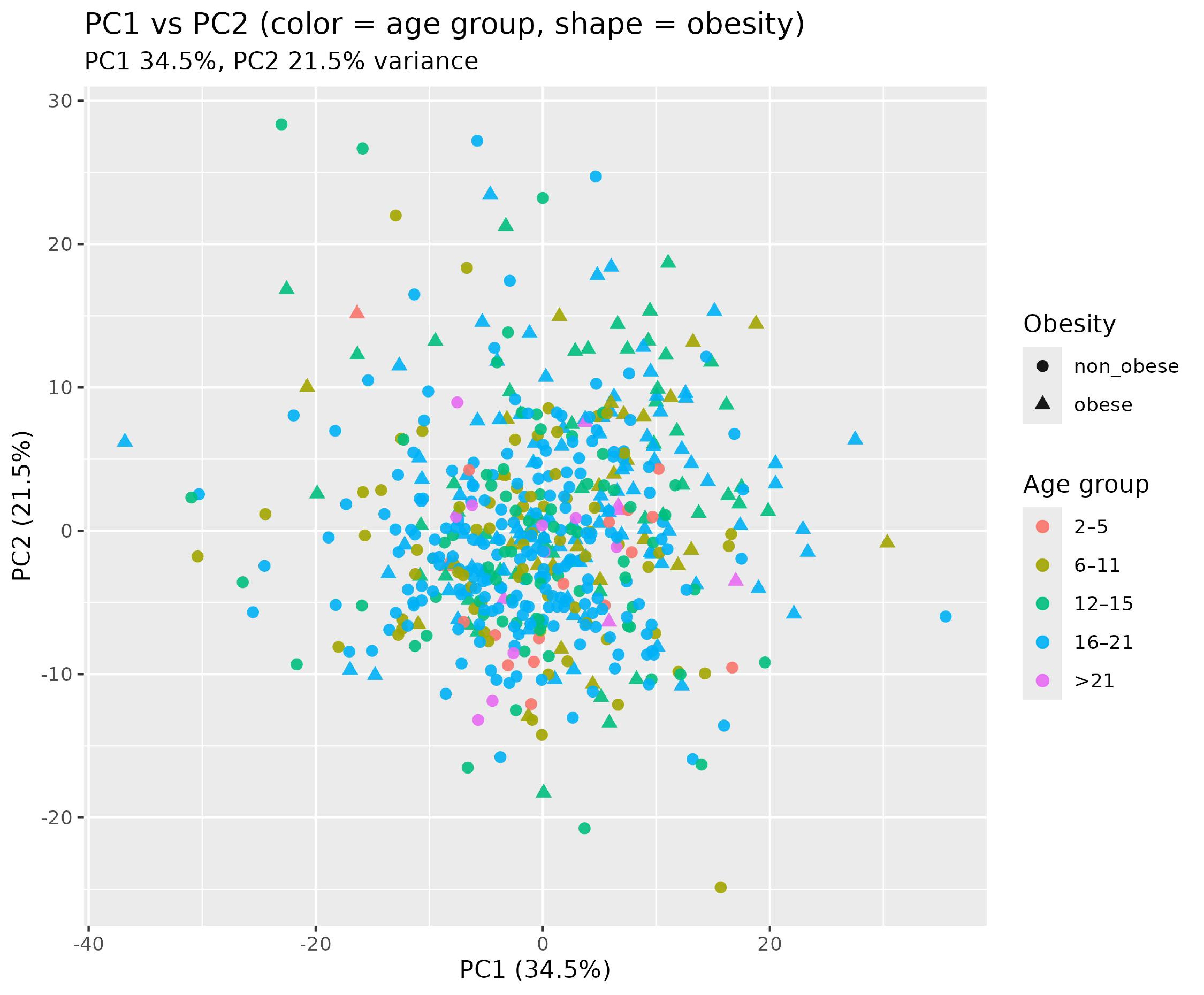
PC1 vs PC2 (color = age group, shape = obesity)
PC1 34.5%, PC2 21.5% variance
PC2 (21.5%)
30
20
10
0
-10
-20
-40
-20
0
20
PC1 (34.5%)
Obesity
non_obese
obese
Age group
2–5
6–11
12–15
16–21
>21
