# Supplementary material for: Metabolic remodeling and the modulatory role of vitamin D deficiency in African American children and adolescents with obesity
Source: Int J Obes (Lond). 2026 Jan 12;50(4):777–87. doi: 10.1038/s41366-025-02003-0 (PMC13056574; doi:10.1038/s41366-025-02003-0)
Supplement: Supplementary file 4 — Supplementary File S2 [file 41366_2025_2003_MOESM4_ESM.pdf]

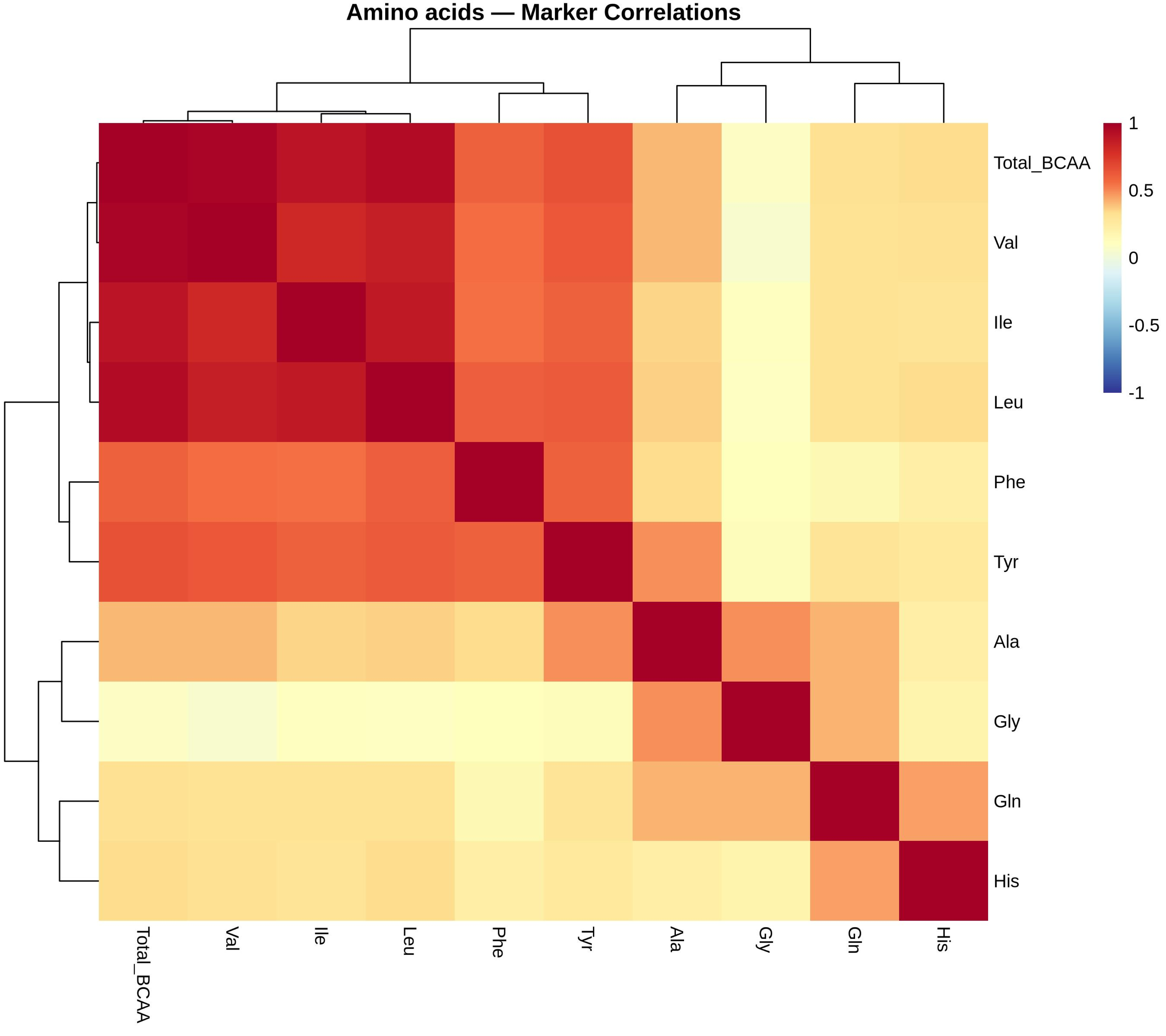

Amino acids — Marker Correlations
Total_BCAA
Val
Ile
Leu
Phe
Tyr
Ala
Gly
Gln
His
1
0.5
0
-0.5
-1

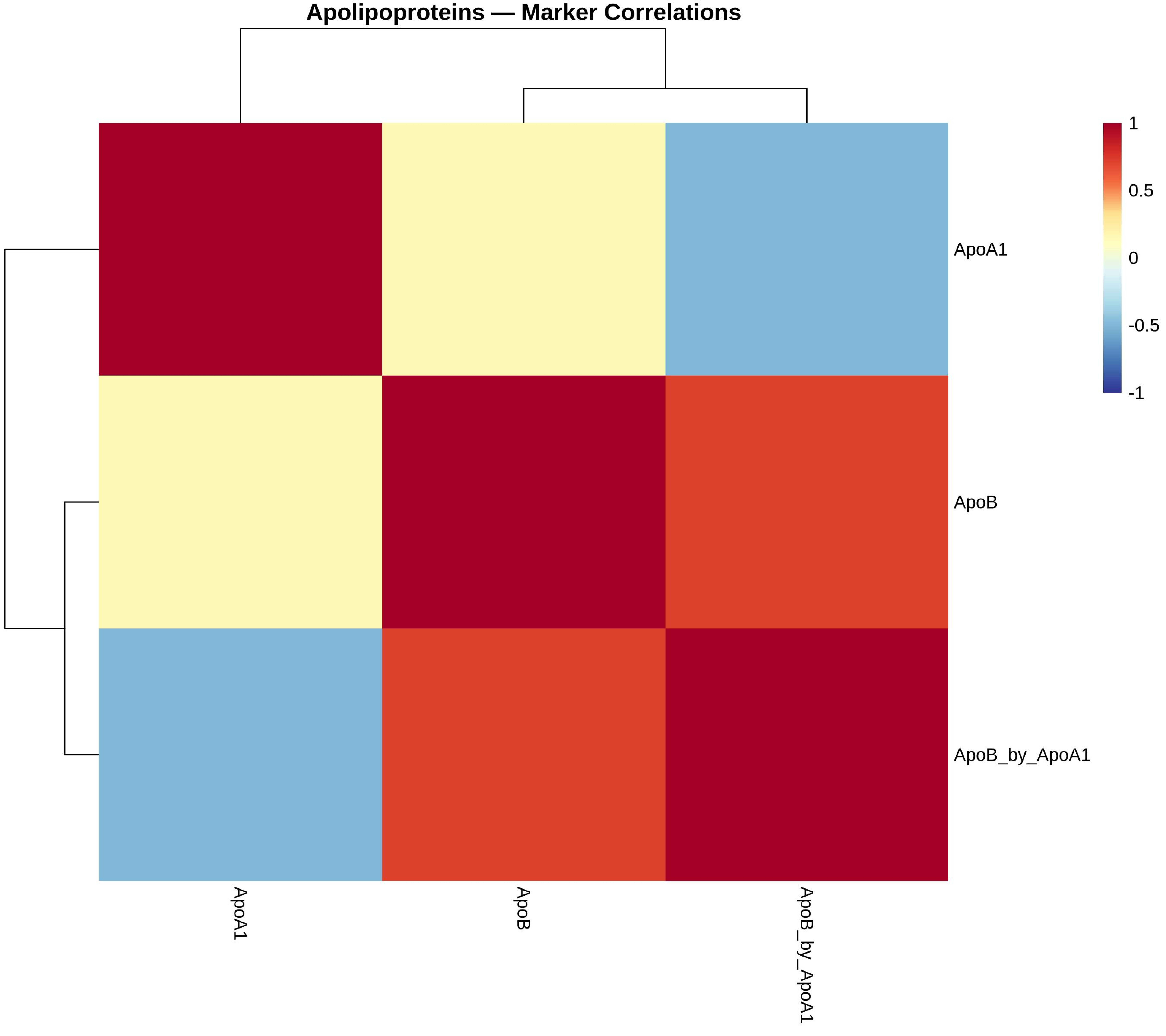
Apolipoproteins — Marker Correlations
ApoA1
ApoB
ApoB_by_ApoA1
ApoA1
ApoB
ApoB_by_ApoA1
1
0.5
0
-0.5
-1

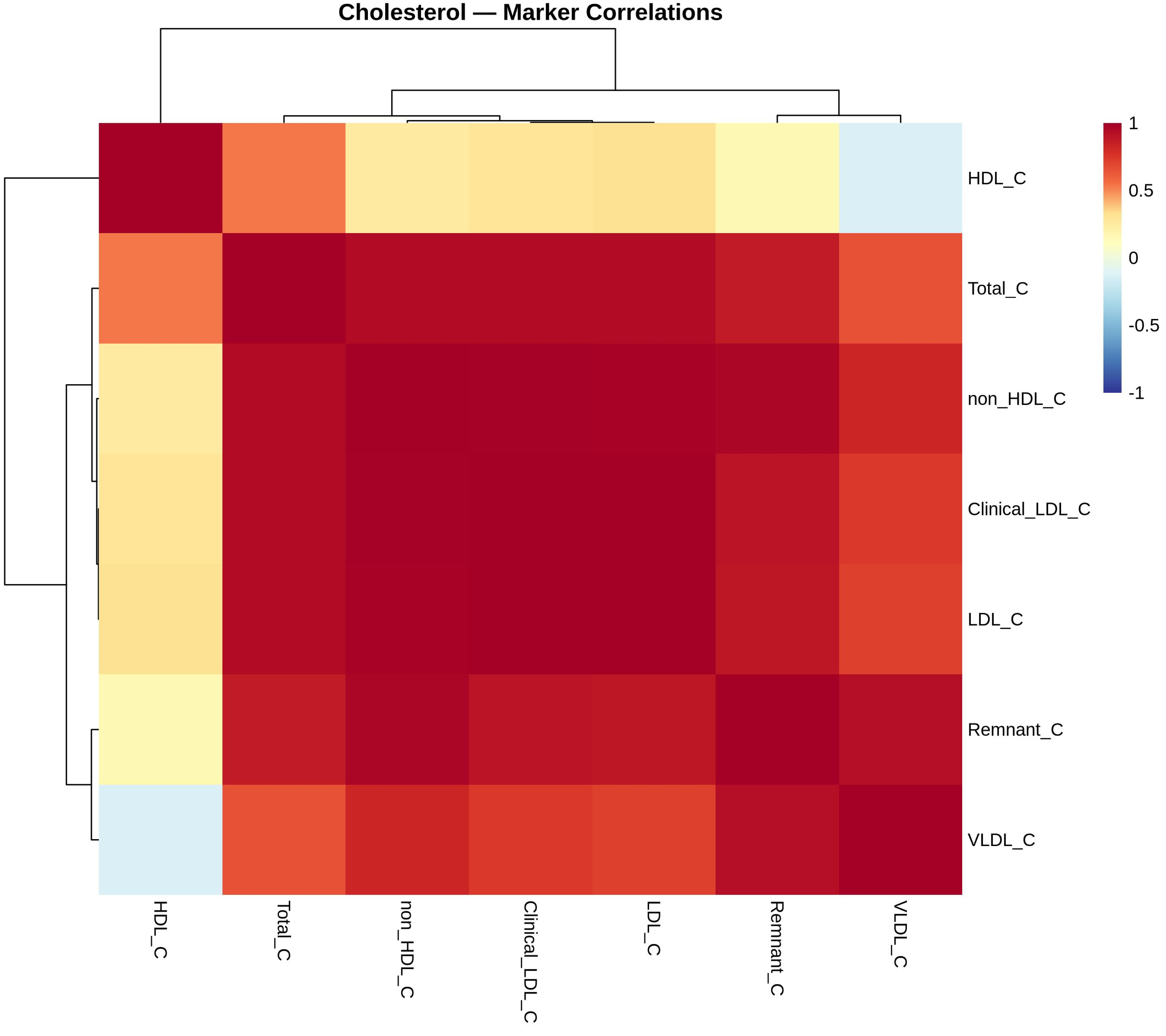

Cholesterol — Marker Correlations
HDL_C
Total_C
non_HDL_C
Clinical_LDL_C
LDL_C
Remnant_C
VLDL_C
1
0.5
0
-0.5
-1

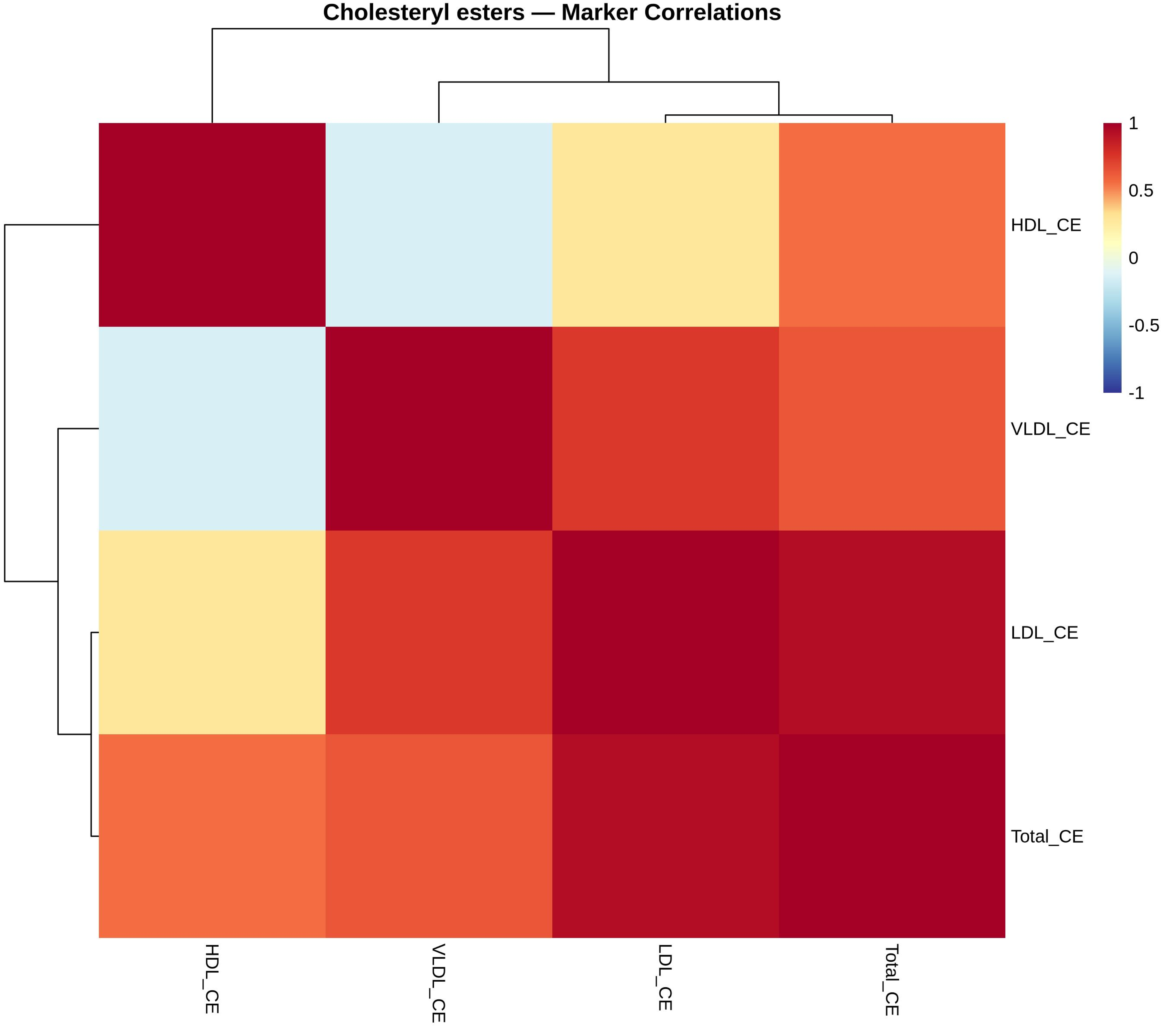

Cholesteryl esters — Marker Correlations
HDL_CE
VLDL_CE
LDL_CE
Total_CE
HDL_CE
VLDL_CE
LDL_CE
Total_CE
1
0.5
0
-0.5
-1

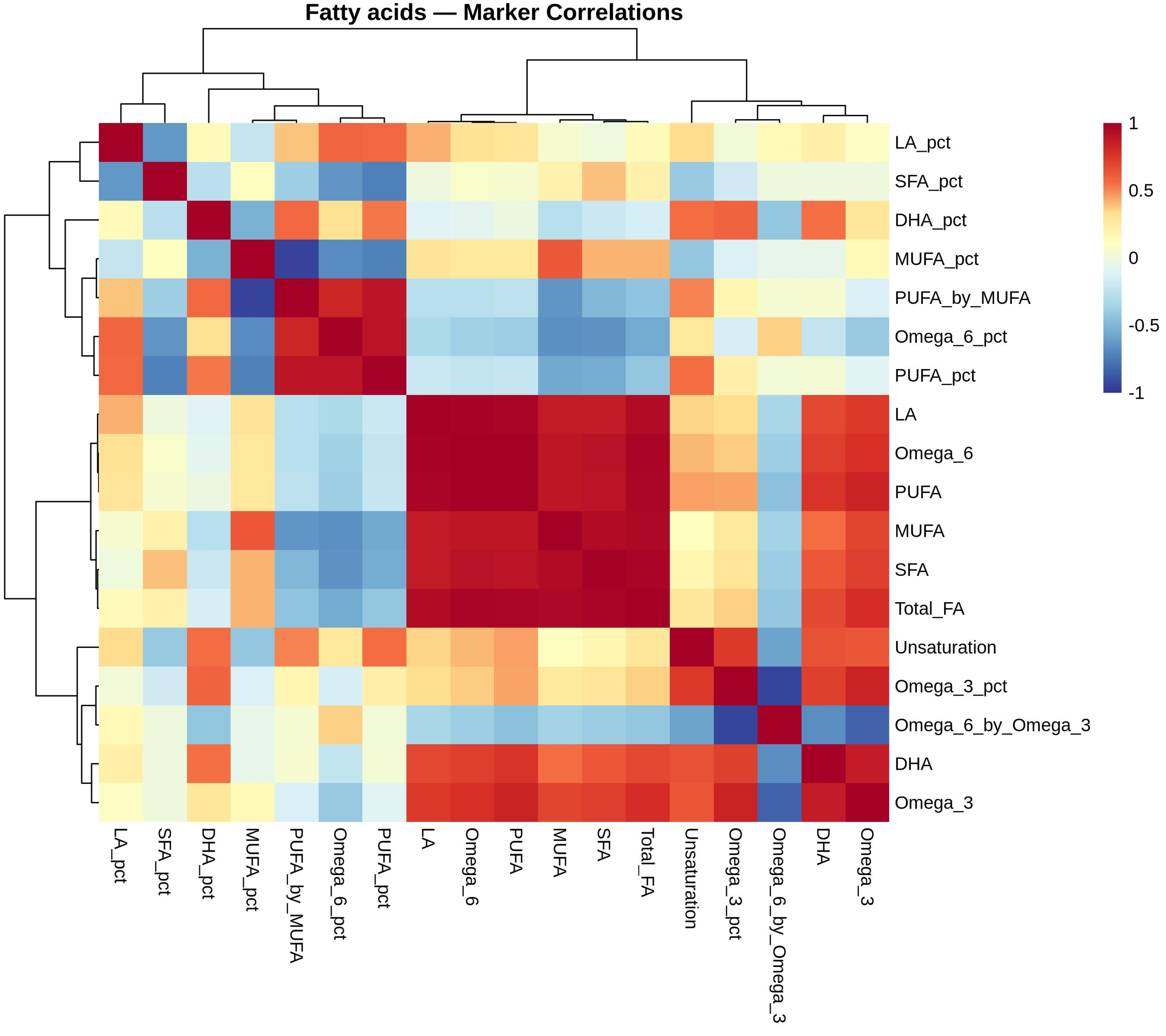
Fatty acids — Marker Correlations
LA_pct
SFA_pct
DHA_pct
MUFA_pct
PUFA_by_MUFA
Omega_6_pct
PUFA_pct
LA
Omega_6
PUFA
MUFA
SFA
Total_FA
Unsaturation
Omega_3_pct
Omega_6_by_Omega_3
DHA
Omega_3
1
0.5
0
-0.5
-1

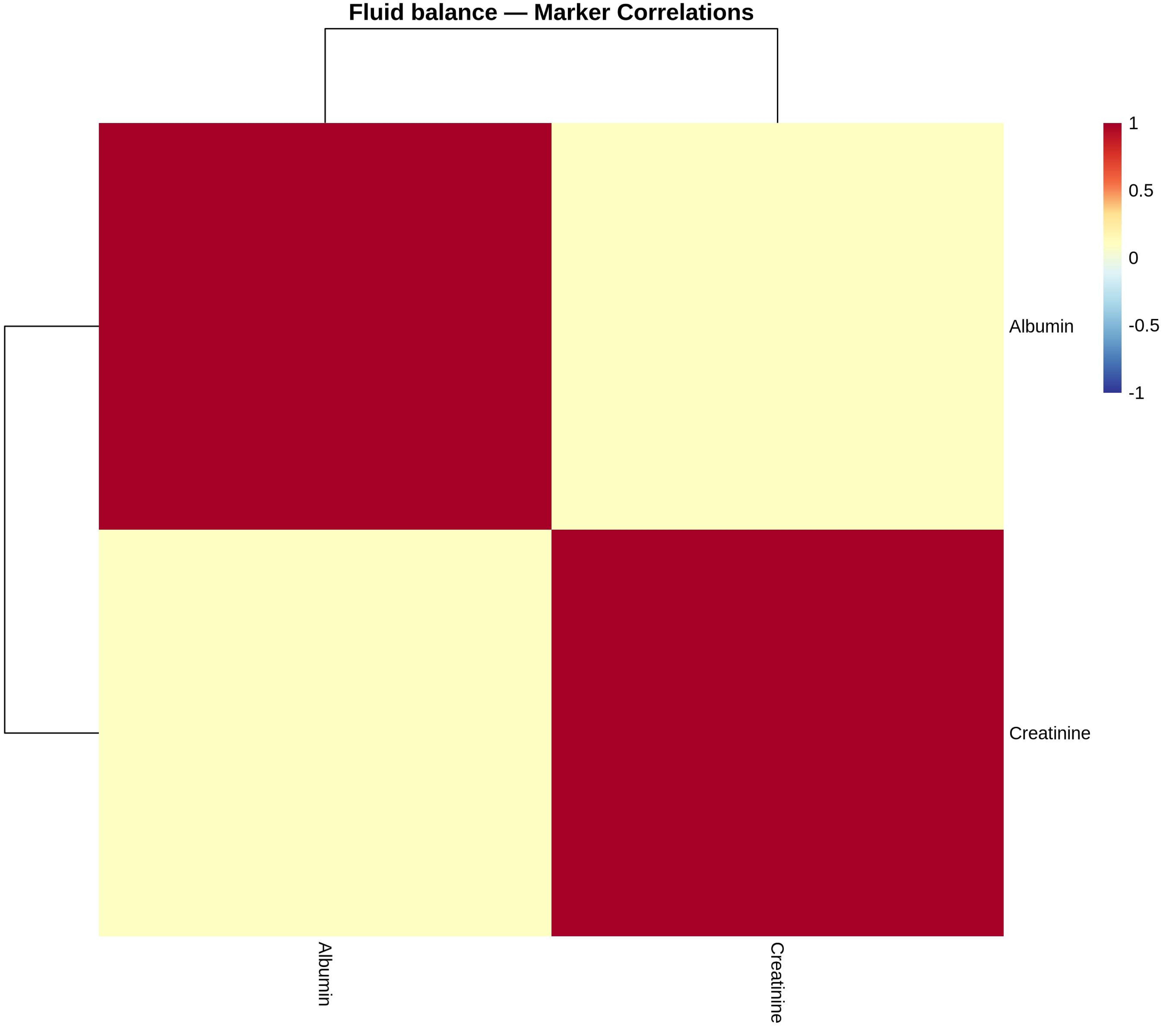
Fluid balance — Marker Correlations
Albumin
Creatinine
Albumin
Creatinine
1
0.5
0
-0.5
-1

Free cholesterol — Marker Correlations

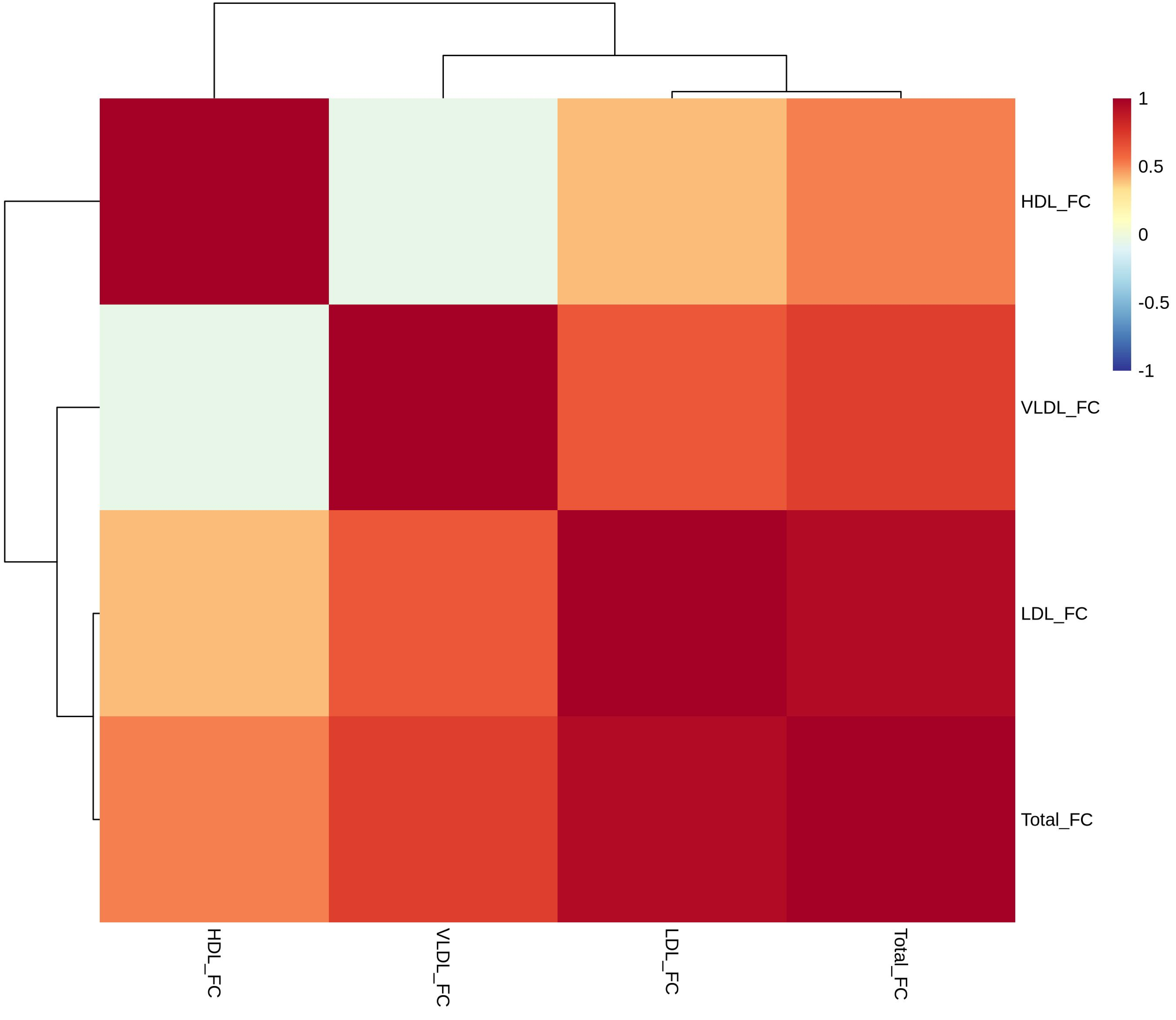

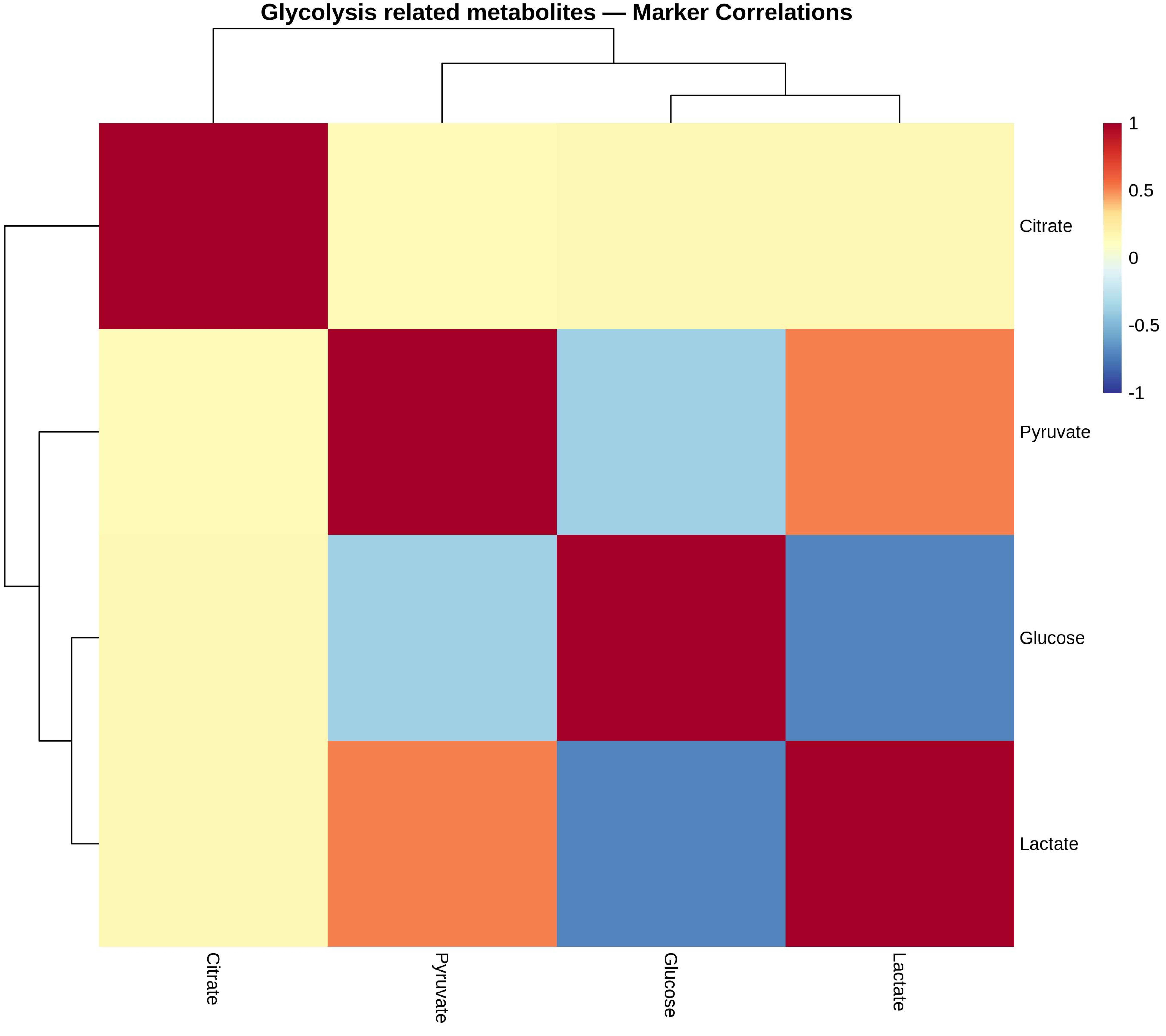

Glycolysis related metabolites — Marker Correlations
Citrate
Pyruvate
Glucose
Lactate
1
0.5
0
-0.5
-1

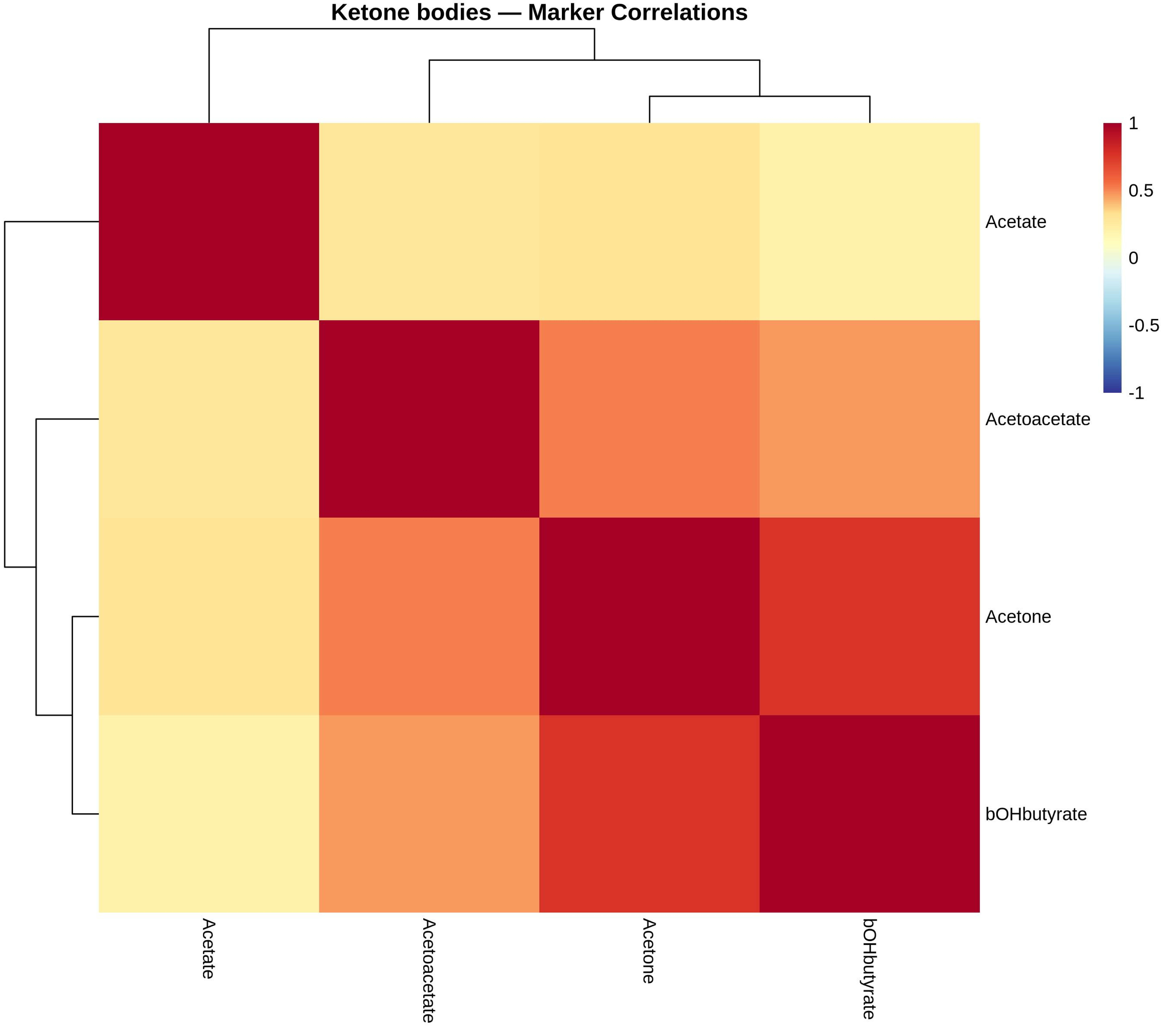
Ketone bodies — Marker Correlations
1
0.5
0
-0.5
-1
Acetate
Acetoacetate
Acetone
bOHbutyrate
Acetate
Acetoacetate
Acetone
bOHbutyrate

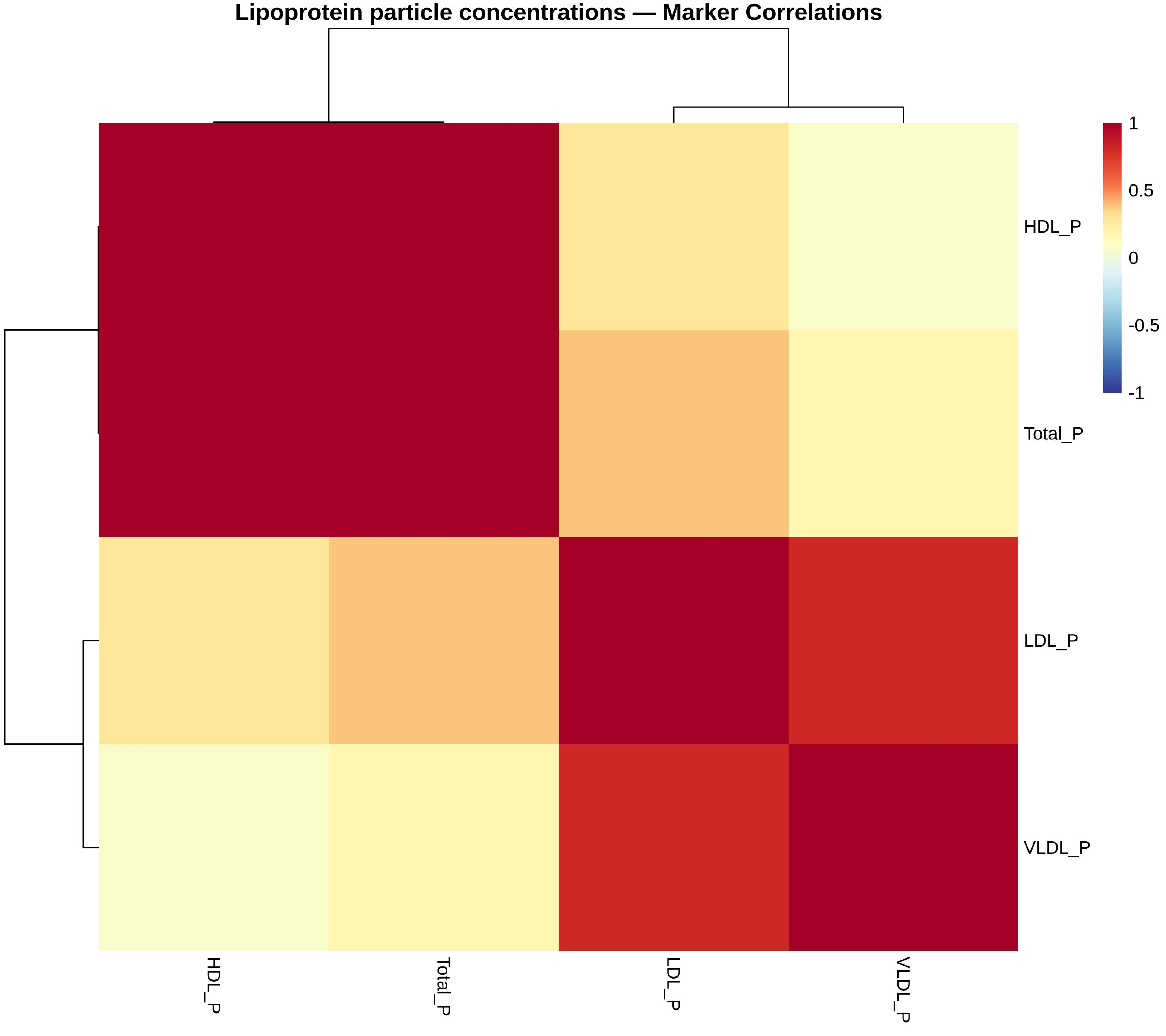
Lipoprotein particle concentrations — Marker Correlations
HDL_P
Total_P
LDL_P
VLDL_P
1
0.5
0
-0.5
-1

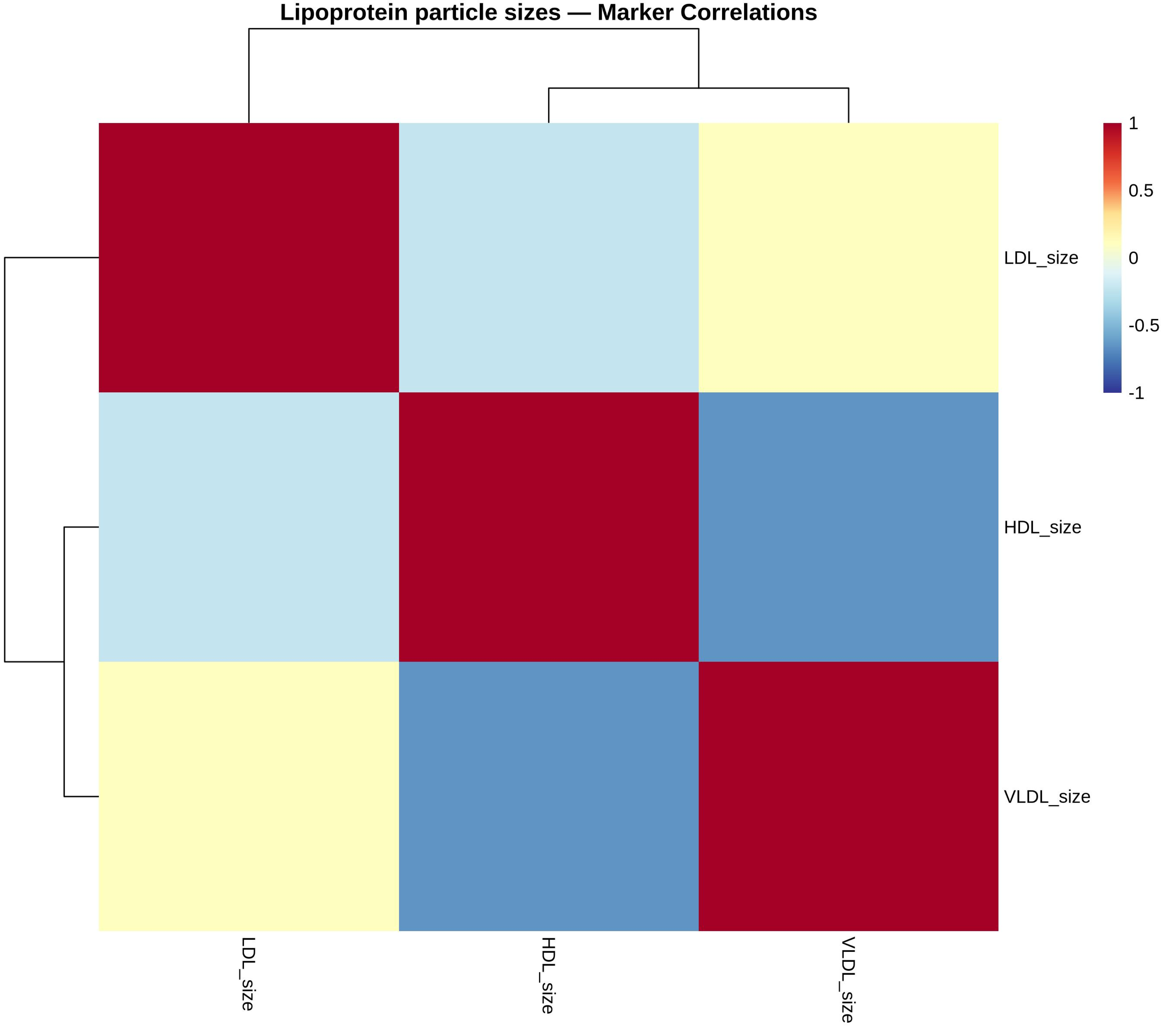
Lipoprotein particle sizes — Marker Correlations
LDL_size
HDL_size
VLDL_size
LDL_size
HDL_size
VLDL_size
1
0.5
0
-0.5
-1

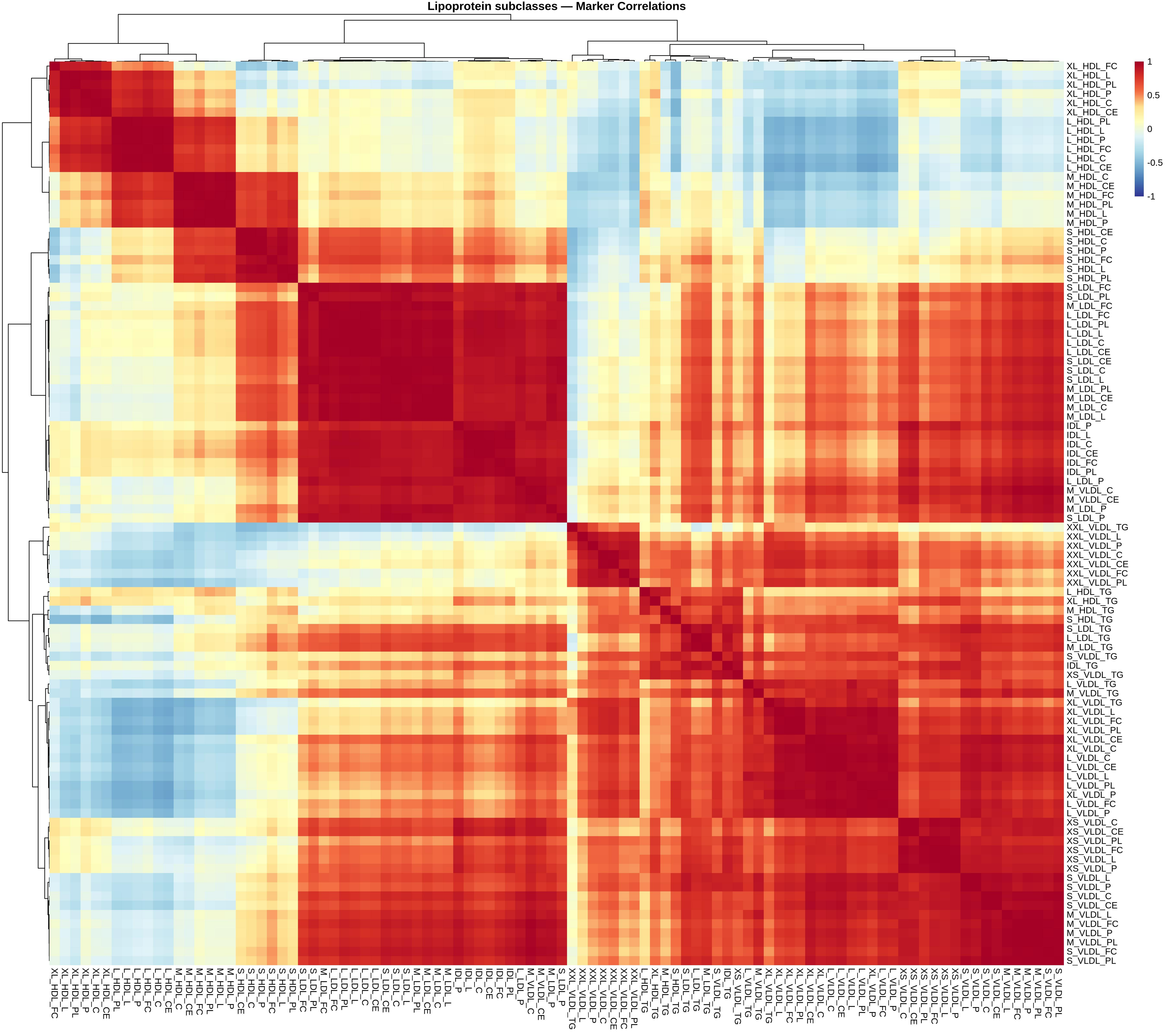

Lipoprotein subclasses — Marker Correlations
1
0.5
0
-0.5
-1
XL_HDL_FC
XL_HDL_L
XL_HDL_PL
XL_HDL_P
XL_HDL_C
XL_HDL_CE
L_HDL_PL
L_HDL_L
L_HDL_P
L_HDL_FC
L_HDL_C
L_HDL_CE
M_HDL_C
M_HDL_CE
M_HDL_FC
M_HDL_PL
M_HDL_L
M_HDL_P
S_HDL_CE
S_HDL_C
S_HDL_P
S_HDL_FC
S_HDL_L
S_HDL_PL
S_LDL_FC
S_LDL_PL
M_LDL_FC
L_LDL_FC
L_LDL_PL
L_LDL_L
L_LDL_C
L_LDL_CE
S_LDL_CE
S_LDL_C
S_LDL_L
M_LDL_PL
M_LDL_CE
M_LDL_C
M_LDL_L
IDL_P
IDL_L
IDL_C
IDL_CE
IDL_FC
IDL_PL
L_LDL_P
M_VLDL_C
M_VLDL_CE
M_LDL_P
S_LDL_P
XXL_VLDL_TG
XXL_VLDL_L
XXL_VLDL_P
XXL_VLDL_C
XXL_VLDL_CE
XXL_VLDL_FC
XXL_VLDL_PL
L_HDL_TG
XL_HDL_TG
M_HDL_TG
S_HDL_TG
S_LDL_TG
L_LDL_TG
M_LDL_TG
S_VLDL_TG
IDL_TG
XS_VLDL_TG
L_VLDL_TG
M_VLDL_TG
XL_VLDL_TG
XL_VLDL_L
XL_VLDL_FC
XL_VLDL_PL
XL_VLDL_CE
XL_VLDL_C
L_VLDL_C
L_VLDL_CE
L_VLDL_L
L_VLDL_PL
XL_VLDL_P
L_VLDL_FC
L_VLDL_P
XS_VLDL_C
XS_VLDL_CE
XS_VLDL_PL
XS_VLDL_FC
XS_VLDL_L
XS_VLDL_P
S_VLDL_L
S_VLDL_P
S_VLDL_C
S_VLDL_CE
M_VLDL_L
M_VLDL_FC
M_VLDL_P
M_VLDL_PL
S_VLDL_FC
S_VLDL_PL

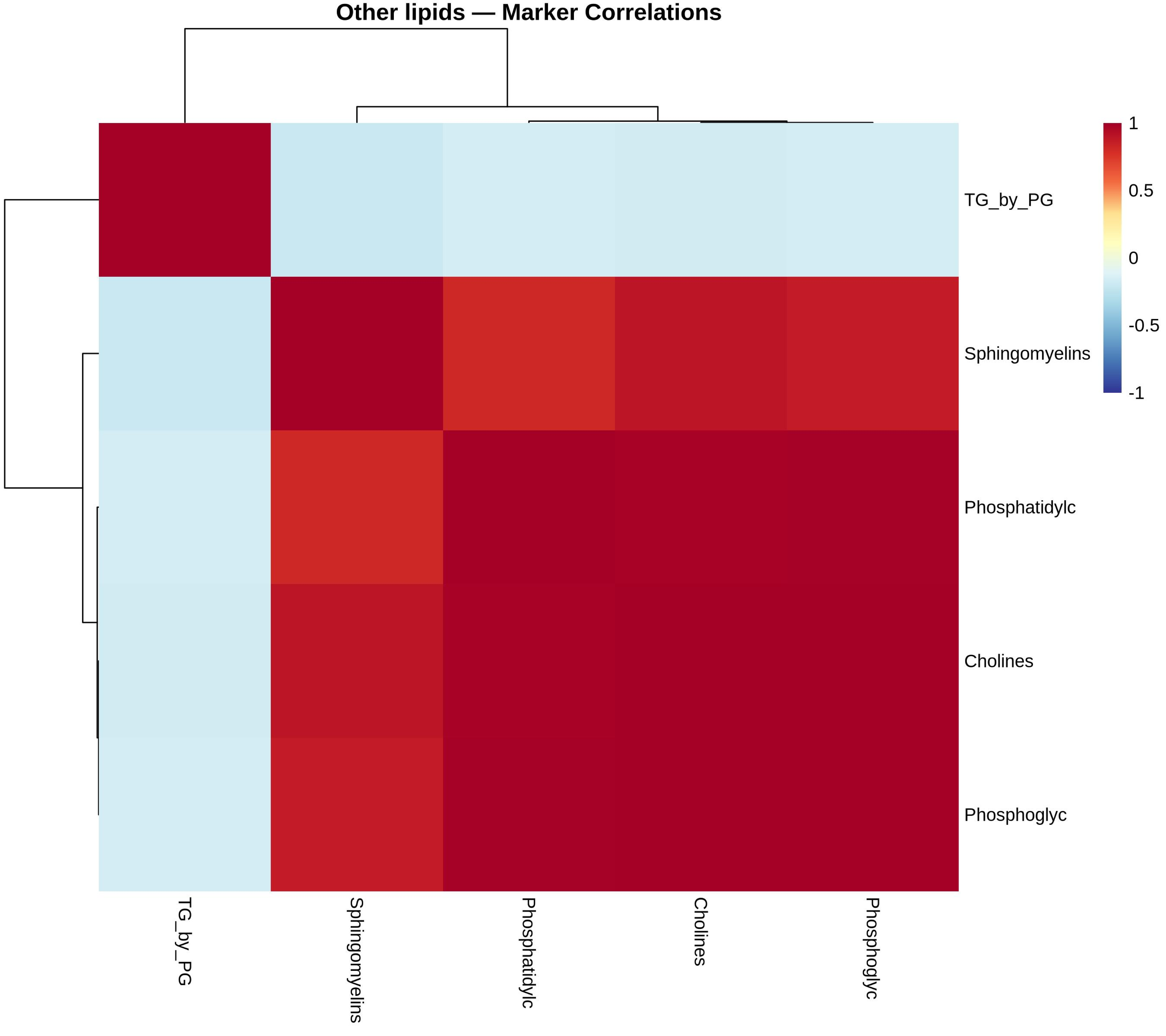
Other lipids — Marker Correlations
TG_by_PG
Sphingomyelins
Phosphatidylc
Cholines
Phosphoglyc
1
0.5
0
-0.5
-1

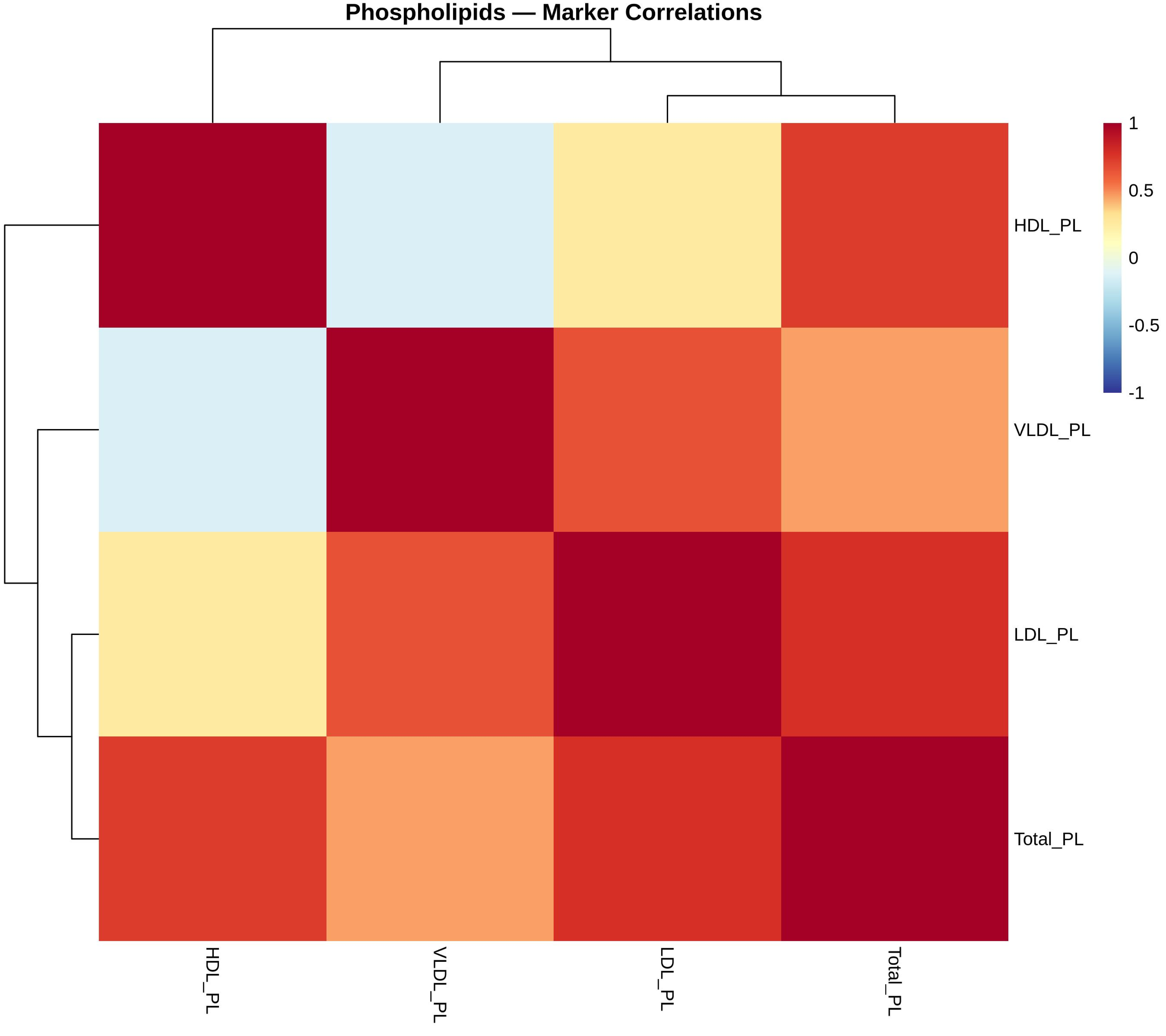

Phospholipids — Marker Correlations
HDL_PL
VLDL_PL
LDL_PL
Total_PL
1
0.5
0
-0.5
-1

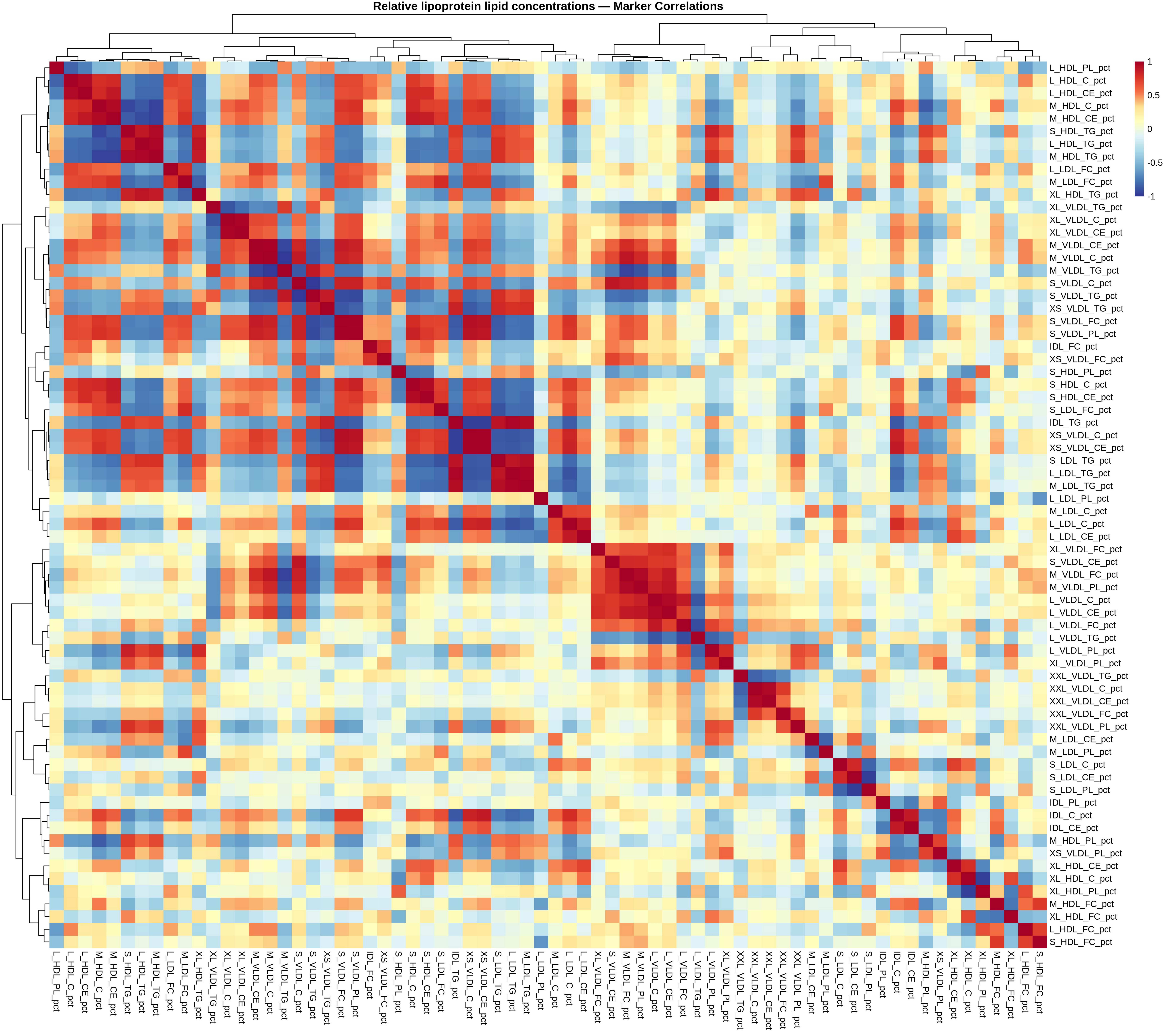

Relative lipoprotein lipid concentrations — Marker Correlations
1
0.5
0
-0.5
-1
L_HDL_PL_pct
L_HDL_C_pct
L_HDL_CE_pct
M_HDL_C_pct
M_HDL_CE_pct
S_HDL_TG_pct
L_HDL_TG_pct
M_HDL_TG_pct
L_LDL_FC_pct
M_LDL_FC_pct
XL_HDL_TG_pct
XL_VLDL_TG_pct
XL_VLDL_C_pct
XL_VLDL_CE_pct
M_VLDL_CE_pct
M_VLDL_C_pct
M_VLDL_TG_pct
S_VLDL_C_pct
S_VLDL_TG_pct
XS_VLDL_TG_pct
S_VLDL_FC_pct
S_VLDL_PL_pct
IDL_FC_pct
XS_VLDL_FC_pct
S_HDL_PL_pct
S_HDL_C_pct
S_HDL_CE_pct
S_LDL_FC_pct
IDL_TG_pct
XS_VLDL_C_pct
XS_VLDL_CE_pct
S_LDL_TG_pct
L_LDL_TG_pct
M_LDL_TG_pct
L_LDL_PL_pct
M_LDL_C_pct
L_LDL_C_pct
L_LDL_CE_pct
XL_VLDL_FC_pct
S_VLDL_CE_pct
M_VLDL_FC_pct
M_VLDL_PL_pct
L_VLDL_C_pct
L_VLDL_CE_pct
L_VLDL_FC_pct
L_VLDL_TG_pct
L_VLDL_PL_pct
XL_VLDL_PL_pct
XXL_VLDL_TG_pct
XXL_VLDL_C_pct
XXL_VLDL_CE_pct
XXL_VLDL_FC_pct
XXL_VLDL_PL_pct
M_LDL_CE_pct
M_LDL_PL_pct
S_LDL_C_pct
S_LDL_CE_pct
S_LDL_PL_pct
IDL_PL_pct
IDL_C_pct
IDL_CE_pct
M_HDL_PL_pct
XS_VLDL_PL_pct
XL_HDL_CE_pct
XL_HDL_C_pct
XL_HDL_PL_pct
M_HDL_FC_pct
XL_HDL_FC_pct
L_HDL_FC_pct
S_HDL_FC_pct

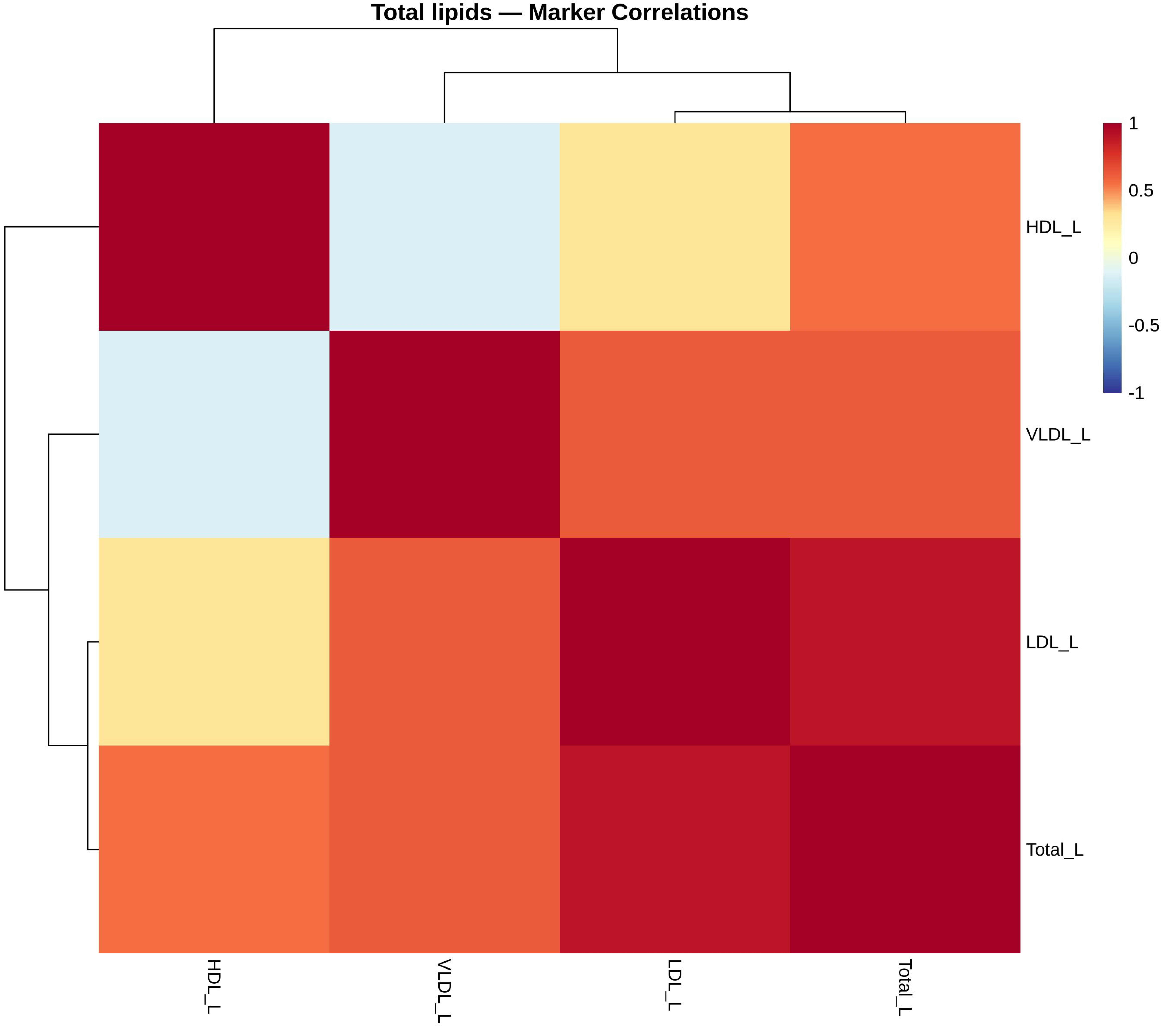

Total lipids — Marker Correlations
1
0.5
0
-0.5
-1
HDL_L
VLDL_L
LDL_L
Total_L
HDL_L
VLDL_L
LDL_L
Total_L

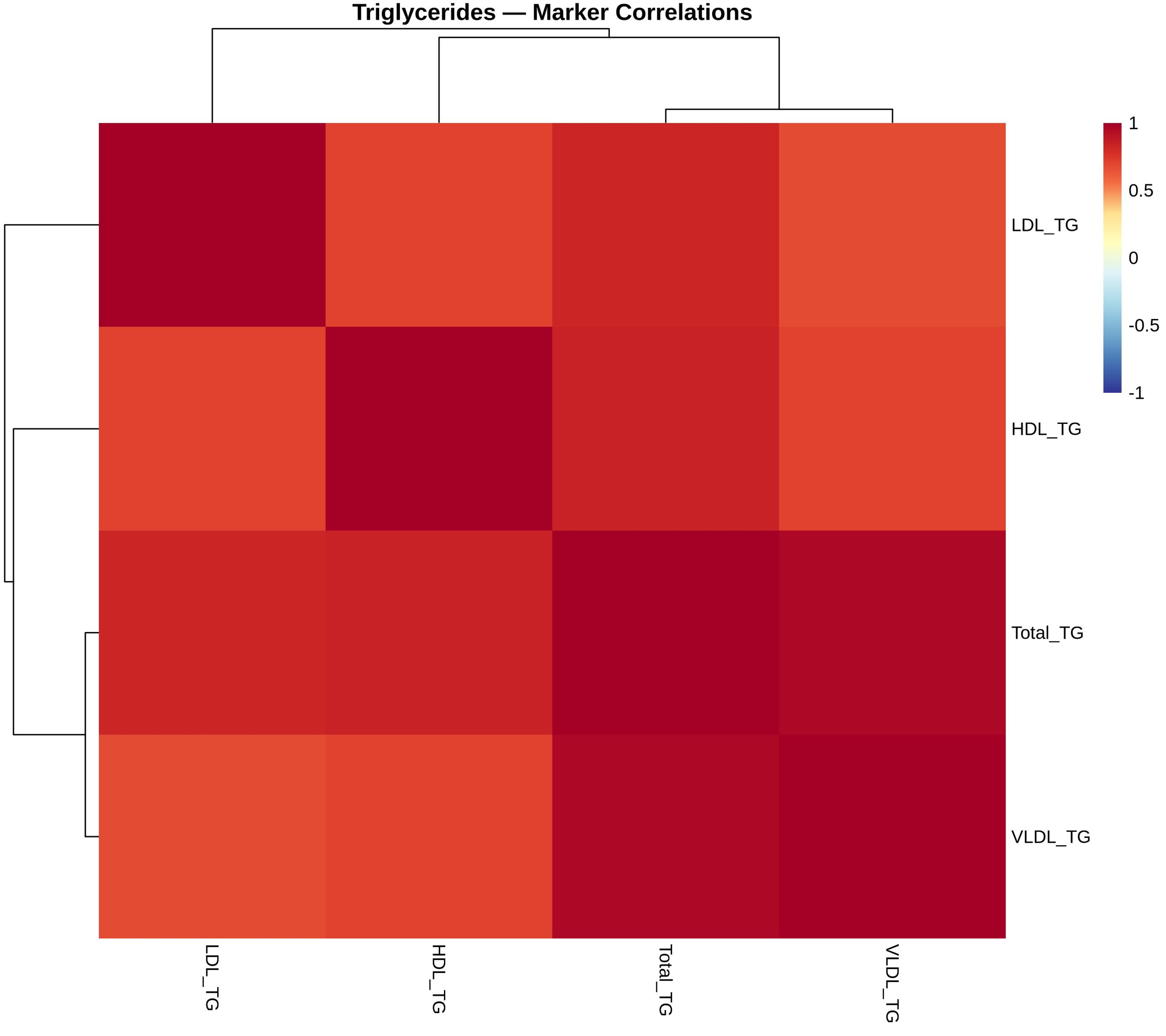
Triglycerides — Marker Correlations
LDL_TG
HDL_TG
Total_TG
VLDL_TG
LDL_TG
HDL_TG
Total_TG
VLDL_TG
1
0.5
0
-0.5
-1
